# Plasmonic tuning of dark-exciton radiation dynamics and far-field emission directionality in monolayer WSe_2_

**DOI:** 10.1126/sciadv.aea5781

**Published:** 2026-01-16

**Authors:** Shuaiyu Jin, Feihong Liu, Ilya Razdolski, Tsz Wing Lo, Yaorong Wang, Zhiwei Peng, Kuan Liang, Ye Zhu, Wang Yao, Anatoly V. Zayats, Dangyuan Lei

**Affiliations:** ^1^ Department of Materials Science and Engineering, City University of Hong Kong, Hong Kong S.A.R. 999077, China.; ^2^ Photonics Research Centre, Universiti Malaya, 50603 Kuala Lumpur, Malaysia.; ^3^ Department of Physics and London Centre for Nanotechnology, King’s College London, Strand, London WC2R 2LS, UK.; ^4^ Department of Applied Physics, Research Institute for Smart Energy, The Hong Kong Polytechnic University, Hung Hom, Hong Kong S.A.R. 999077, China.; ^5^ New Cornerstone Science Laboratory, Department of Physics, The University of Hong Kong, Hong Kong S.A.R. 999077, China.; ^6^ Hong Kong Institute of Quantum Science & Technology, The University of Hong Kong, Hong Kong S.A.R. 999077, China.; ^7^ Department of Physics, Centre for Functional Photonics, Hong Kong Branch of National Precious Metals Material Engineering Research Centre, and Hong Kong Institute of Clean Energy, City University of Hong Kong, Hong Kong S.A.R. 999077, China.

## Abstract

Manipulation of excitonic emission properties is important for numerous photonic applications. Of particular interest are developing easy-to-implement yet effective approaches for controlling the radiation dynamics and directionality of spin-forbidden dark excitons (X_D_) in two-dimensional semiconductors. Here, we investigate the spectral, temporal, and directional characteristics of room-temperature X_D_ emission from a tungsten diselenide monolayer coupled to a dissipative plasmonic nanocavity. Under resonant plasmon-exciton coupling, the radiative decay rate of X_D_ is accelerated by nearly four orders of magnitude, and correspondingly, the X_D_ lifetime is shortened to a subnanosecond level, making it comparable to that of bright excitons. Fitting the measured lifetimes with a Purcell-formalism–based cavity quantum electrodynamics model allows estimating of the intrinsic room-temperature X_D_ lifetime to be about 24 ± 2.3 microseconds. Furthermore, the measured radiation patterns of the dark excitons show that subtle variations in the nanocavity orientation can effectively tailor the X_D_ emission directionality, important for quantum technologies and optoelectronics applications.

## Teaser

Plasmonic nanocavities enable spectral, temporal, and directional control over the dark-exciton radiation in monolayer WSe_2_.

## INTRODUCTION

In monolayer transition metal dichalcogenides (ML TMDCs), the lack of inversion symmetry and strong spin-orbit coupling result in spin splitting of the conduction band, typically on the order of a few tens of milli–electron volt. This leads to the spin-valley coupling of electronic bands at the K and K′ valleys (*1*, *2*). Within each spin-split conduction band, electrons with opposite spins can interact with holes in the corresponding valence bands through Coulomb attraction. These interactions give rise to spin-allowed bright excitons (X_O_) and spin-forbidden dark excitons (X_D_) (*3*, *4*). Group theory analysis has demonstrated that the transition dipole orientations of X_O_ and X_D_ are perpendicular to each other (*5*, *6*). In addition, X_D_ exciton exhibits a significantly longer lifetime compared to X_O_ exciton due to the selection rules because the optical transition of X_D_ exciton requires a spin-flip process (*7*, *8*). Previous research has indicated that the lifetime of X_D_ excitons is two orders of magnitude longer than that of X_O_ ones at cryogenic temperature (~10 K) (*9*). However, the lifetime difference between X_D_ and X_O_ at room temperature remains unclear, as X_D_ exciton emission is typically undetectable under these conditions.

The rate of excitonic transitions is governed by both the dipole strength of the optical transition and the local density of optical states, as described by Fermi’s golden rule. Accordingly, two principal strategies have emerged to enhance X_D_ emission. The first one involves enabling a nonzero dipole moment *D* of the relevant optical transition through a magnetic field–induced spin mixing (*6*, *10*–*12*). The second approach focuses on increasing the local density of optical states by coupling the excitonic emitter to optical cavities. Plasmonic nanostructures have been shown to markedly accelerate forbidden transitions in molecules, exploiting associated strongly inhomogeneous fields (*12*), and this mechanism can be used for controlling X_D_ emission in two-dimensional (2D) materials. The emission of X_D_ can be enhanced by coupling with either propagating surface plasmon polaritons on a metal film at cryogenic temperatures (*13*) or localized surface plasmons (LSP) in nanocavities formed by a tip (*8*) or a nanoparticle-on-mirror structure (*14*). Both approaches have already paved the way for further applications of X_D_ excitons in nanoscale devices. However, the underlying mechanism and characteristics of both magnetic field–induced and plasmon-enhanced X_D_ emission remain unexplored, not to mention the possibility of further manipulating its properties.

In this work, we use a class of ultracompact gold nanocube-on-mirror (NCoM) cavities with tunable plasmon resonance to manipulate the room-temperature emission properties of X_D_ excitons in monolayer WSe_2_, focusing on the corresponding radiation dynamics and directionality. We observe a giant X_D_ emission enhancement from the WSe_2_-NCoM hybrid structure and a marked reduction of the room-temperature X_D_ lifetime to a subnanosecond level, comparable to that of the spin-allowed X_O_ (~450 ps). Tuning the energy difference between X_D_ exciton and the nanocavity gap-plasmon mode enables systematic manipulation of the X_D_ emission intensity and lifetime. Using a quantum electrodynamics model based on the Purcell formalism for dissipative cavities to fit the measured lifetimes, we estimate the intrinsic room-temperature lifetime of X_D_ to be 24 ± 2.3 μs, corresponding to a Purcell factor of 3 × 10^4^ for the hybrid structure. Back-focal-plane (BFP) images of the X_D_ emission coupled to nanocavities reveal a series of highly distorted donut-shaped patterns, resulting from the redistribution of Purcell factor hotspots within the nanoscale cube-mirror gap (induced by slight tilting of the nanocavity) and the spatially dependent radiation patterns of emitters across the nanogap. The obtained results hold promise in advancing the applications of spin-forbidden X_D_ in quantum technologies and nanoscale optoelectronics (*15*, *16*).

### Results, design, and characterization of coupled WSe_2_-NCoM nanocavities

**Fig. 1.**
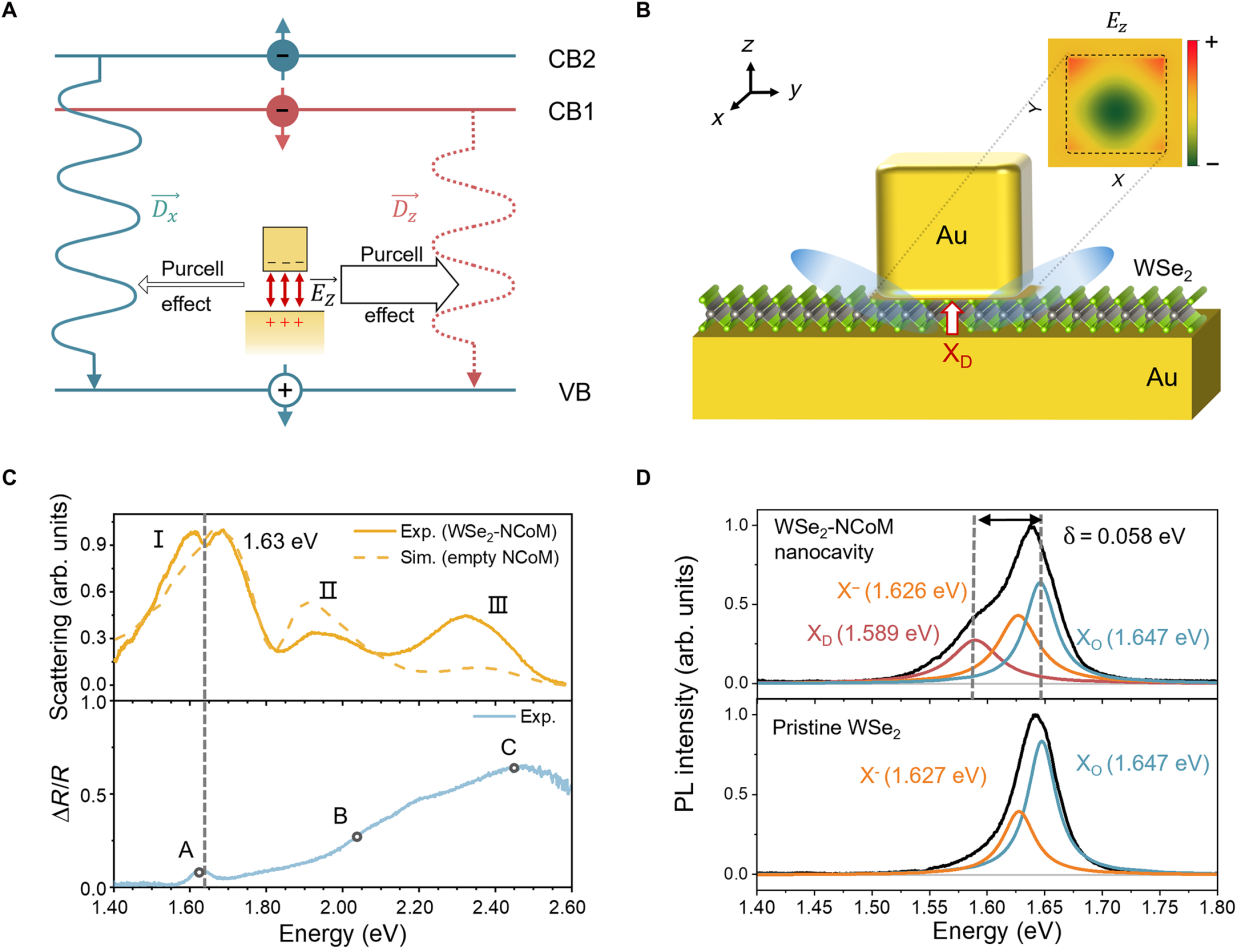
NCoM nanocavity–induced emission of X_D_. (**A**) Energy band diagram of ML WSe_2_ at the K valley. As indicated by the red and blue arrows, electrons in the lower (CB1) and higher (CB2) conduction bands have opposite spins, giving rise to an optically bright exciton state with an in-plane dipole moment (X_O_) and an optically dark exciton state with an out-of-plane dipole moment (X_D_). VB indicates the upper valence band. (**B**) Schematic illustration of the WSe_2_-NCoM cavity. The red arrow represents the out-of-plane X_D_ dipole, which is coupled with the vertical gap plasmon mode of NCoM. The inset shows the calculated distribution of an out-of-plane component of the electric field in NCoM at the gap plasmon resonance (~1.65 eV). (**C**) Top: Dark-field scattering spectrum (solid line) of a single WSe_2_-NCoM cavity under white light illumination. The dashed line shows the simulated scattering spectra of the NCoM cavity without WSe_2_ ML. The dip at around 1.65 eV in the WSe_2_-NCoM cavity spectra originates from the weak coupling between the gap plasmon mode and the X_O_ exciton state in WSe_2_. Bottom: A spectrum of differential reflectance $\frac{\Delta R}{R}=\frac{R_{2}-R_{1}}{R_{2}}$ of a pristine WSe_2_ ML, where *R*_1_ and *R*_2_ are the reflectance values of the Au mirror with and without the WSe_2_ ML, respectively. A, B, and C indicate the energies of bright exciton states. arb., arbitrary; Exp., experiment; Sim., simulation. (**D**) Top: Photoluminescence (PL) spectrum measured under the 532-nm excitation from a single WSe_2_-NCoM cavity (black), overlaid with three Lorentzian fit curves showing the presence of X_O_ (blue), X^−^-trion (orange), and X_D_ (red) emission. Bottom: PL spectra of a pristine WSe_2_ ML, overlaid with two Lorentzian fit curves showing the presence of (blue) X_O_ and (orange) X^−^-trion emission only.

A WSe_2_ monolayer was placed in an ultracompact (gap width ~ 1.8 nm) plasmonic NCoM cavity formed by a gold film and a gold nanocube (Fig. 1B; see Materials and Methods for the details of the fabrication). The related cavity plasmon mode features a strongly confined out-of-plane electric field that couples to dipole moments of the excitons (Fig. 1A).

The dark-field scattering spectrum reveals three plasmonic resonance modes of the WSe_2_-NCoM hybrid system: the out-of-plane gap plasmon mode I centered at ~1.60 eV; another out-of-plane mode II centered at ~1.93 eV with a much stronger localized electric field centered at ~1.93 eV, which makes it inapplicable for efficient resonant coupling with the X_D_ exciton (*17*); and the in-plane transverse mode III centered at ~2.35 eV (Fig. 1C). The inset in Fig. 1B shows the distribution of the out-of-plane electric field component for mode I. This field *E_z_* is notably stronger than the in-plane components and asymmetric due to oblique incidence (fig. S3). By optimizing the nanocube size (~85-nm edge length) and the Au mirror thickness (~100 nm), we can tune the resonance energy of the gap plasmon mode I to the energy of X_D_ (~1.59 eV). Furthermore, the scattering spectrum of a WSe_2_-NCoM shows a dip in the vicinity of mode I, which, according to the differential reflectivity data of a bare WSe_2_ monolayer, can be attributed to the weak coupling between the X_O_ and the resonant gap plasmon mode with the resulting spectrum being the addition of the individual spectra of the cavity and the exciton, which are slightly shifted with respect to each other (Fig. 1C) (*18*, *19*).

The emission spectrum of WSe_2_-NCoM is dominated by the prominent bright exciton emission peak centered at ~1.65 eV (Fig. 1D), accompanied by a broad shoulder in the lower energy range. Similar results were obtained on another sample where the WSe_2_ layer outside of the cavity was removed by reactive ion etching (fig. S5), thus confirming the pivotal role of the NCoM cavity coupling for the X_D_ emission. In contrast, the photoluminescence (PL) spectrum of the bare WSe_2_ monolayer shows the emission from the bright excitons X_O_ and trions X^−^ without additional notable features (Fig. 1D). The PL spectrum in the top panel of Fig. 1D uses three Lorentzians corresponding to the X_O_ (blue), trion (orange), and X_D_ (red) exciton states. Notably, the X_O_ and trion energies closely match the respective exciton energies in the bare WSe_2_ monolayer. The energy difference between X_O_ and X_D_ is ~58 meV, in agreement with previous reports (*9*, *14*). The fitting results show that the X_D_ linewidth (~54 meV) is larger than that of the X_O_ (~32 meV), which might be attributed to the inhomogeneous contribution brought by the gold film (*20*) and the plasmon-exciton coupling–induced decrease in the PL quantum yield (*21*).

**Fig. 2.**
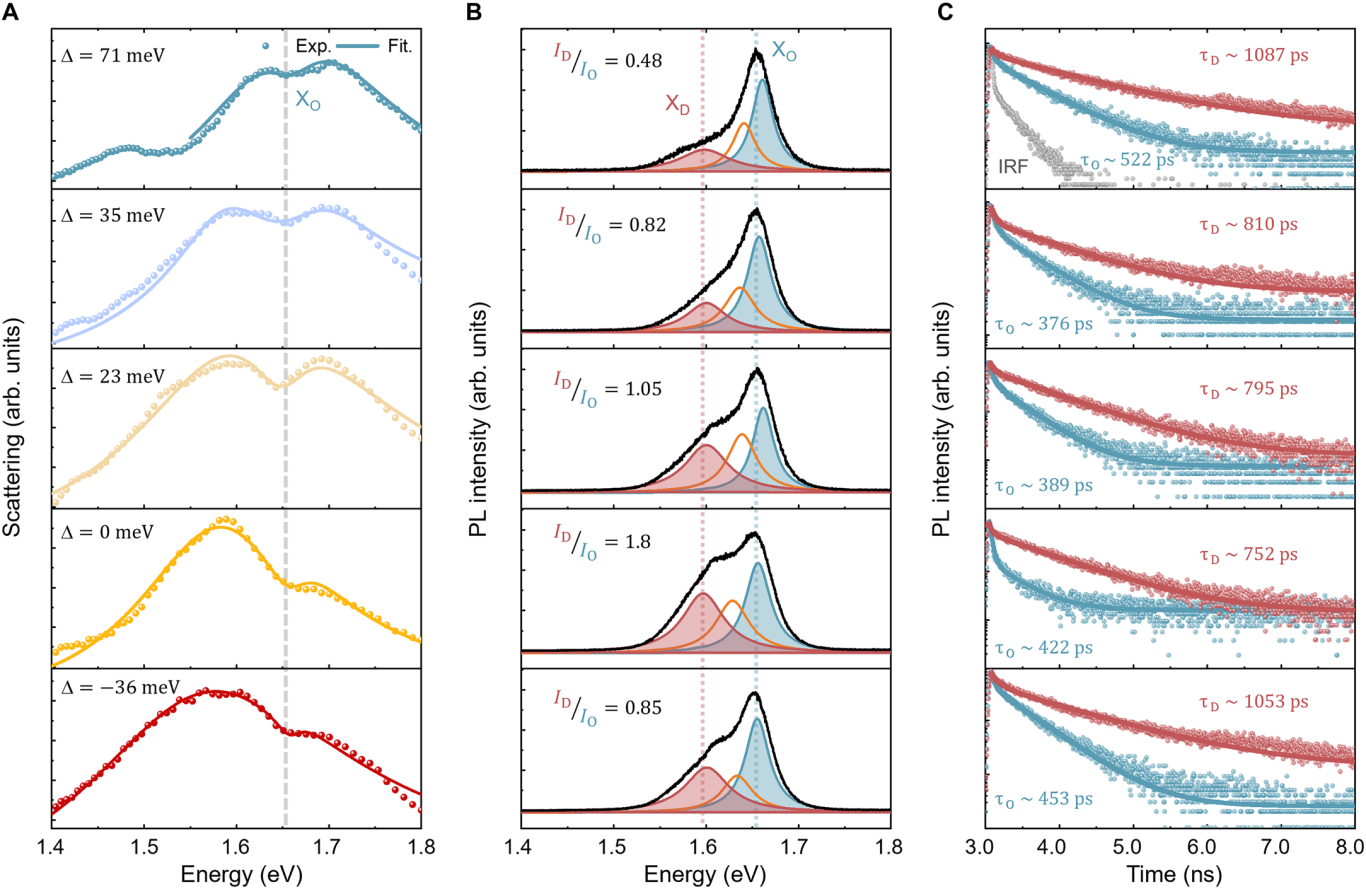
Dark-field scattering, PL, and TRPL responses of WSe_2_-NCoM nanocavities. (**A**) Dark-field scattering spectra for different gap plasmon mode resonances as indicated by various detuning (D) between the energy of the gap plasmon mode (*E*_P_) and the energy of the X_D_ exciton (*E*_D_). Symbols represent experimental results, and lines are fitting results by a coupled-oscillator model. The gray dashed line indicates the energy of bright excitons. Fit., fitting. (**B**) Corresponding PL spectra, overlaid with three Lorentzian fit curves. $\frac{I_{D}}{I_{O}}$ is the peak area ratio of X_D_ to X_O_. The red (blue) dotted line represents the peak energy of X_D_ (X_O_) exciton. (**C**) Corresponding TRPL responses of X_D_ (red dots) and X_O_ excitons (blue dots). Gray dots are the instrument response function (IRF). The solid lines represent biexponential fitting.

Further insights into the emission properties of the nanocavities can be obtained by tuning the LSP resonance away from the exciton band. To understand the effect of spectral detuning and its impact on the intensities and lifetime of X_D_ exciton, we examined 14 WSe_2_-NCoM nanocavities (fig. S8). Figure 2A shows dark-field scattering spectra of five representative WSe_2_-NCoM nanocavities, each exhibiting a different energy detuning between the plasmon mode and X_D_ resonance. The detuning was achieved through the subtle size variations of individual nanocubes, resulting in the different resonance frequencies of mode I in Fig. 1C. The detuning energy can be estimated from the scattering spectra using the fitting with the coupled oscillator model (*22*). The dispersion of X_O_ and the plasmon mode extracted from the model are shown in fig. S9, revealing no avoided crossing (Rabi splitting). The corresponding PL spectra are shown in Fig. 2B, with three Lorentzian fits used to extract the peak areas of X_D_, X^−^, and X_O_. The peak area ratio of X_D_ to X_O_ emission first increases and then decreases, as the gap plasmon mode shifts from red detuned to blue detuned relative to the X_D_ energy. Similar trends were observed at low temperature (150 K; fig. S10). Time-resolved PL (TRPL) measurements in Fig. 2C reveal the lifetimes of X_D_ and X_O_ excitons at ~1 ns and 500 ps, respectively. Notably, the X_D_ lifetime shows a nonmonotonic trend, first decreasing and then increasing, as the detuning changes from red to blue relative to the X_D_ one. In contrast, the X_O_ lifetime remains nearly constant across all detuning conditions. Also, the linewidths of X_D_ and X_O_ excitons exhibit distinct trends, as the energy between the gap plasmon mode and excitons is tuned, confirming the coupling between X_D_ and the gap plasmon mode (fig. S11).

### Estimation of the intrinsic room-temperature lifetime of X_D_

**Fig. 3.**
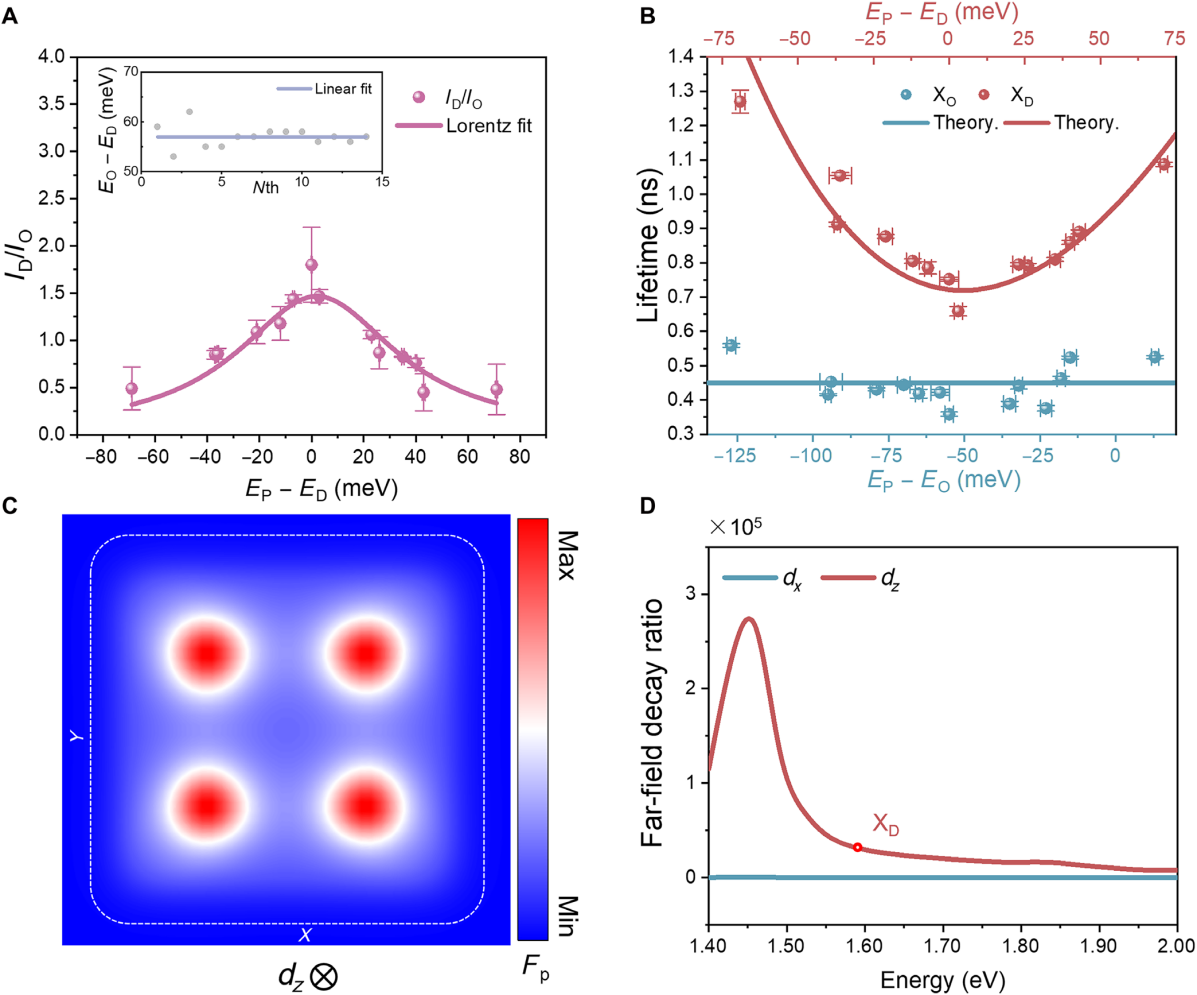
Plasmonic tuning of the dark-exciton emission intensity and radiation dynamics. (**A**) Emission intensity ratio of X_D_ to X_O_ excitons as a function of energy detuning between the gap plasmon mode and the X_D_ resonance. The error bars represent the uncertainty in the parameter estimates. The solid line is the Lorentzian fit. The inset displays the energy splitting between X_O_ and X_D_ excitons across 14 nanocavities, with a mean value of 57 meV and an SD of 2.1 meV. (**B**) Measured lifetimes of the X_D_ (red dots) and X_O_ (blue dots) excitons as a function of energy detuning between the gap plasmon mode and the X_D_ or X_O_ resonances. The error bars for both axes are derived from the uncertainties in the fitting process. The solid line is calculated with Eq. 1. (**C**) Spatial distribution of the radiative decay rate enhancement at 1.59 eV in an NCoM nanocavity. The maximum Purcell factor (*F*p) of ~40,000 is observed at the locations close to the four corners of the nanocube. (**D**) Simulated spectra of the far-field radiative decay rate enhancement $\Gamma_{\text{FF}}/\Gamma_{0}$ for the in-plane (*d_x_*) and out-of-plane (*d_z_*) dipoles in the regions of the maximum Purcell enhancement.

Figure 3A presents the ratio of the peak areas of X_D_ to X_O_ emission as a function of the energy detuning between the gap plasmon mode and X_D_. The trend follows a Lorentzian profile with a linewidth of ~100 meV, closely matching the linewidth of the gap plasmon mode observed in the scattering spectrum (Fig. 1C). The energy splitting between X_O_ and X_D_ excitons is 57 ± 2.1 meV across the nanocavities studied. Notably, the X_D_ and X_O_ lifetimes depend differently on cavity-exciton detuning: As the detuning increases, the X_D_ lifetime is significantly increased, whereas the X_O_ lifetime remains unchanged (Fig. 3B).

To explain the impact of dissipative cavity detuning on the X_D_ lifetime, we consider a cavity quantum electrodynamics model based on the Purcell formalism. Within this framework, when considering the weak coupling between dipole emitters and dissipative nanoresonators, the spontaneous decay rate Γ of an emitter in the cavity can be described as (*23*)

$$\Gamma =F\Gamma_{0}\frac{\omega_{0}^{2}}{\omega^{2}}\frac{\omega_{0}^{2}}{\omega_{0}^{2}+4Q^{2}{\left(\omega -\omega_{0}\right)}^{2}}\left[1+2Q\frac{\omega -\omega_{0}}{\omega_{0}}\frac{\text{Im}\left(\tilde{V}\right)}{\text{Re}\left(\tilde{V}\right)}\right]$$ (1)

where ω is the emitter frequency, $\omega_{0}$ is the resonant frequency of the cavity, and $\Gamma_{0}$ is the intrinsic X_D_ spontaneous decay rate in a bare WSe_2_ ML. The quality factor of the cavity Q is defined as $\omega_{0}/\Delta \omega$ with Δω being the bandwidth of the cavity and $\tilde{V}$ being the mode volume of the cavity, calculated accounting for dissipation (*24*). In addition, F signifies the generalized Purcell factor, which is determined as

$$F=\frac{3}{4\pi^{2}}{\left(\frac{\lambda_{0}}{n}\right)}^{3}\text{Re}\left(\frac{Q}{\tilde{V}}\right)$$ (2)

where $\lambda_{0}$ is the X_D_ wavelength in the WSe_2_ and n is the refractive index of a WSe_2_ ML (*25*). Using the electromagnetic field distributions in the cavity calculated using the finite element method (see Materials and Methods for details), for the parameters of the NCoM used in the experiment, we obtain the following: $\frac{\tilde{V}}{\lambda_{0}^{3}}=\left(1.93-0.02i\right)\times 10^{-7}$, $Q\approx 10,F\approx 3.3\times 10^{4}$, and thus $\Gamma_{0}\approx \left(4.2\pm 0.4\right)\times 10^{-5}\;{\text{ns}}^{-1}$. These estimates provide an excellent agreement with the experimental data (Fig. 2B). The error bar of $\Gamma_{0}$ indicates the variability of $\Gamma_{0}$ derived from fitting to the experimental data (fig. S14). On the basis of this, we deduce that the X_D_ lifetime in a bare WSe_2_ ML at room temperature is ~24 ± 2.3 μs. To the best of our knowledge, this is the first reported estimation of the intrinsic radiative lifetime of X_D_ at room temperature.

The quasi-parabolic relationship between X_D_ lifetime and cavity detuning, described by Eq. 1, agrees well with the experimental findings obtained from multiple nanocavities (Fig. 3B). The optimal enhancement wavelength, at which the shortest lifetime is observed, is blue shifted (~5 meV) relative to the exciton emission energy as seen from the fit. This blue shift can be predominantly attributed to dissipation-induced spectral shifts in the NCoM cavity (see note S3 for detailed analysis). In addition, other factors may contribute, including the intrinsic blue shift between the exciton absorption and emission energies (*26*) and the typical blue shift of near-field resonance relative to far-field scattering in strongly coupled nanocavities (*27*). It is also important to consider that these experiments are inevitably affected by inhomogeneous broadening arising from variations in gap morphology and inherent local characteristics of WSe_2_, leading to fluctuations across different samples. Moreover, the orthogonal polarizations of X_D_ and X_O_ dipoles result in markedly different mode volumes and thus the Purcell factors. Hence, the cavity-driven enhancement of the X_O_ emission is highly inefficient (Fig. 3B), resulting in the negligible dependence of the X_O_ lifetime on the detuning energy. A slight asymmetry in lifetime between positive and negative detuning was also observed, an expected feature of dissipative nanocavities (*23*). Notably, our findings reveal that dissipation is pivotal in the intricate dependency of X_D_ lifetime on detuning. Nevertheless, this NCoM cavity allows the reduction of the X_D_ lifetime down to ~650 ps (four orders of magnitude) while maintaining the intrinsic X_O_ lifetime at 444 ± 57 ps.

To investigate the spatial distribution of the Purcell factor inside the cavity, we used the MNPBEM toolbox (*28*) to conduct the numerical simulations and compute the Purcell factor at the X_D_ energy (1.59 eV). A pronounced enhancement of the Purcell factor for out-of-plane dipole polarization was observed (Fig. 3C). The total decay rate Γ (fig. S13) is composed of both near-field and far-field components: $\Gamma =\Gamma_{\text{FF}}+\Gamma_{\text{NF}}$. When placing a dipole in the region of the maximum Purcell enhancement, the far-field decay rate as a function of the emission wavelength was simulated, showing different trends for the emitters with in-plane and out-of-plane polarization directions (Fig. 3D). The maximum intensity is observed around the X_D_ position, indicating a decay rate enhancement larger than 7000. Considering the fluctuations in the size and shape of the colloidal nanocubes, which can result in variations in the plasmon resonances of the WSe_2_-NCoM cavities, it may be possible to achieve even larger on-resonance local density of optical states and radiation efficiency. This could further enhance the X_D_ emission rate. Compared to the in-plane dipole, the far-field radiative efficiency of the out-of-plane dipole in the NCoM cavity is much stronger (fig. S13). This also demonstrates the potential to overcome the intrinsic weak transition dipole moment of X_D_ excitons and manipulate their emission and dynamics at room temperature.

### Directionality control of X_D_ radiation by nanocavity configurations

**Fig. 4.**
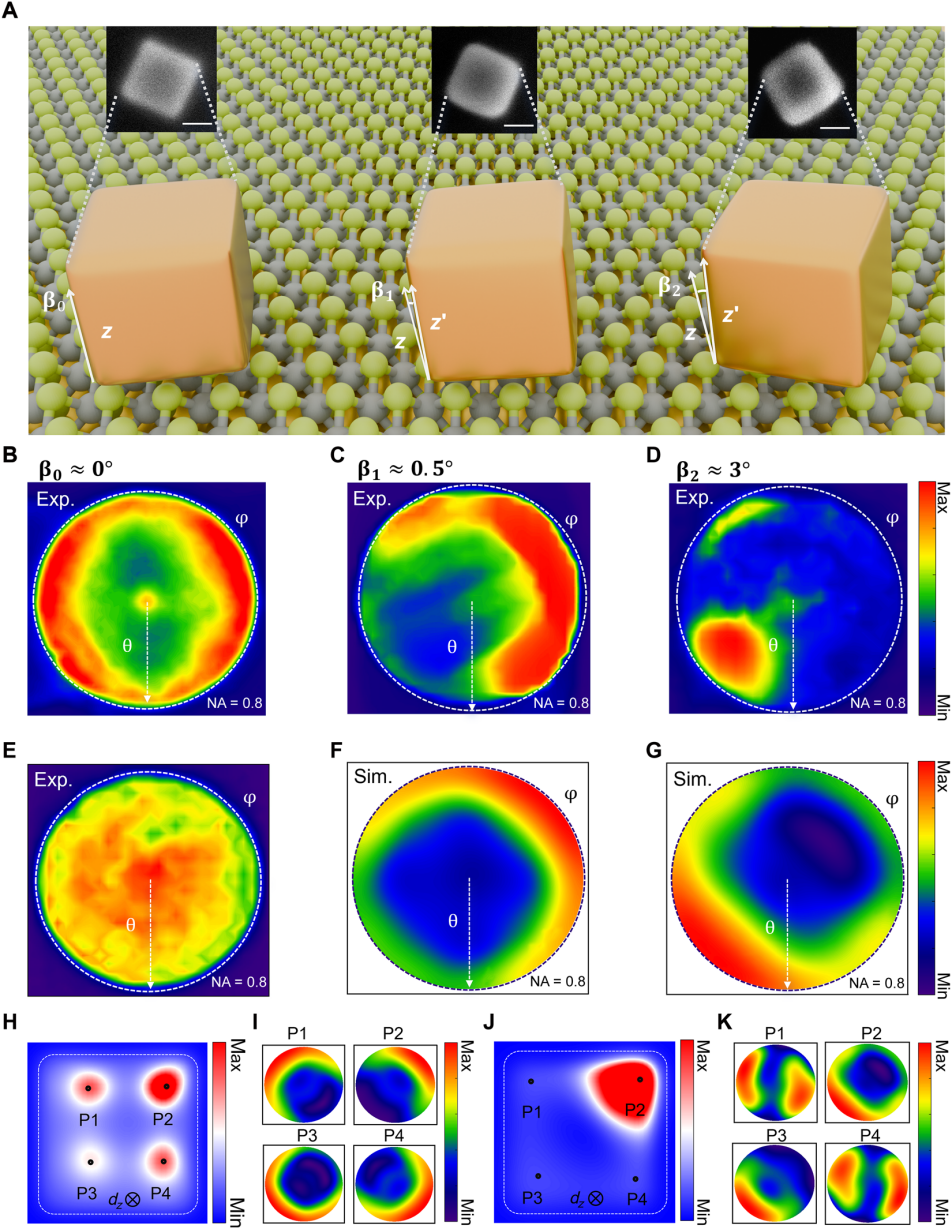
Plasmonic tuning of the dark-exciton emission directionality. (**A**) Schematic illustration of three WSe_2_-NCoM nanocavities with different orientations, where $\beta_{o}$, $\beta_{1}$, and $\beta_{2}$ denote the angles between the *z* axis and the normal (*z*′) of the top surface of each nanocube. Insets show SEM micrographs of the respective WSe_2_-NCoM cavities. Scale bars, 50 nm. (**B** to **D**) BFP images of X_D_ radiation from three WSe_2_-NCoM nanocavities: (A) for $\beta_{0}\approx 0{}^{\circ}$, (B) for $\beta_{1}\approx 0.5{}^{\circ}$, and (C) for $\beta_{2}\approx 3{}^{\circ}$, showing distinct patterns correlated with the nanocube orientation in Fig. 4A. The white dotted line represents the maximum angular collection range of the objective used in the experiments [numerical aperture (NA) = 0.8]. (**E**) Experimentally measured BFP image of X_O_ from WSe_2_-NCoM nanocavities, showing a distinctly different pattern than for X_D_ exciton. (**F** and **G**) Simulated BFP images of X_D_ radiation for two different tilt angles, (F) for $\beta_{1}\approx 0.5{}^{\circ}$ and (G) for $\beta_{2}\approx 3{}^{\circ}$, accounting for the spatially redistributed Purcell factor within the plasmonic gap and the emitter radiation patterns in the corresponding hotspot positions. (**H**) Spatial distributions of the radiative decay rate enhancement at 1.59 eV in the NCoM nanocavity with tilt angle $\beta_{1}\approx 0.5{}^{\circ}$. P1 to P4 correspond to the positions of maximum Purcell factor located near the four corners of the nanocube. (**I**) Simulated BFP images for vertically oriented dipole emitters placed at the four hotspot positions [P1 to P4 in (H)] within the plasmonic gap, each located at a local Purcell factor maximum near a corner. All images are collected using the same objective (NA = 0.8). (**J**) Similar results as (H) but for the tilted angle of $\beta_{2}\approx 3{}^{\circ}$. (**K**) Similar results as (I) but for the tilted angle of $\beta_{2}\approx 3{}^{\circ}$.

NCoM cavities, beyond modulating the radiation rate of excitons, also provide a flexible platform for effectively tailoring the far-field emission directionality of the excitons. Providing a direct visualization of the momentum space distribution, BFP imaging can be used to map the angular distribution of the emitted light (*29*). We first compare the radiation patterns of X_D_ and X_O_ in the WSe_2_-NCoM nanocavity. Figure 4B shows the experimentally measured BFP images of X_D_ from a relatively flat WSe_2_-NCoM nanocavity, which is verified by the corresponding scanning electron microscopy (SEM) micrographs in Fig. 4A, appearing to be a donut-like shape. However, the radiation pattern of X_O_ emission in the WSe_2_-NCoM cavity is pie-like (Fig. 4E), which is barely modified by the gap plasmon mode. Moreover, the X_O_ emission exhibits a one-lobe radiation profile, which is obviously different from that of the donut-like X_D_ radiation pattern (two-lobe profile). The maximum X_D_ PL intensity is observed at ~±36°, whereas the X_O_ PL intensity is maximized at ~0°. The clear difference between the two excitons is seen in fig. S16, where the BFP intensities are azimuthally averaged in the in-plane directions (see Materials and Methods for details), confirming their orthogonal polarizations, with X_D_ oriented out-of-plane and X_O_ aligned in-plane. The 3D simulations of the gap plasmon mode I and transverse mode III also show completely opposite shapes of the radiation patterns, in agreement with the experimental results (fig. S16). These results provide clear evidence for the fundamentally different radiation characteristics of X_D_ and X_O_ excitons in the WSe_2_-NCoM nanocavity.

Because of the plasmon-exciton coupling, the far-field emission directionality of X_D_ is influenced by the plasmon mode geometry. The radiation patterns of X_D_ could be either asymmetric (C) or corner like (D). In particular, the observed asymmetries of X_D_ radiation patterns in Fig. 4 (C and D) can be primarily attributed to the tilt of the nanocavities, which can substantially modify the angular emission profile of embedded dipole emitters (*30*, *31*). The SEM micrographs of the three WSe_2_-NCoM nanocavities in Fig. 4A confirm their different orientations. These cavity-to-cavity variations arise primarily from the inherent randomness of the drop-casting process, along with the surface irregularities of ML WSe_2_ and residual polymer left from the 2D material transfer process, resulting in the varied geometries and tilt angles. In the SEM images, brighter regions indicate higher secondary electron emission, closely related to the escape volume on the surface (fig. S17). This pronounced difference in the radiation patterns fundamentally arises from two main factors. First, even slight tilts result in substantial changes of the Purcell factor distribution (*32*), which exhibits either an asymmetric profile at a slight tilt (Fig. 4H; $\beta_{1}\approx 0.5{}^{\circ}$) or a corner-like pattern at a larger tilt (Fig. 4J; $\beta_{2}\approx 3{}^{\circ}$), with the hotspot shifting closer to the cavity edge as the tilt increases. Second, the far-field radiation patterns of dipoles located at different locations within the plasmonic gap vary markedly (Fig. 4, I and K) (*33*). The interplay of these two effects leads to a marked modulation in the directional emission of X_D_ but with negligible influence on the PL intensity (fig. S15). To accurately capture the overall radiation pattern from the tilted cavity, we consider four representative hotspot positions in the gap (P1 to P4), each corresponding to a local maximum of the Purcell factor near the nanocube corner. The radiation pattern at each point is weighted by its respective Purcell factor, and the sum yields the calculated BFP images shown in Fig. 4 (F and G). The emitter radiation pattern at the dominant Purcell factor hotspot (P2) exhibits a complete reversal between the two different tilt angles (*33*), leading to a distinct overall radiation pattern (Fig. 4, F and G). This demonstrates how subtle changes in nanocube orientation can effectively steer the emission directionality of X_D_ excitons in NCoM cavities, offering a strategy for directional control in nanophotonic systems. To achieve more precise control over its emission directionality, one possible approach is to modify the shape of the top nanoparticles, for example, replacing them with nanodecahedra (*34*) or other sharp-edged polyhedral geometries. These geometries have already been reported to exhibit pronounced directionality in dark-field scattering measurements.

## DISCUSSION

At room temperature, we achieved four orders of magnitude reduction in the PL lifetime of spin-forbidden X_D_ in a WSe_2_ monolayer coupled to a plasmonic nanocavity, making the X_D_ lifetime comparable to that of X_O_. This is because the nanocavity selectively enhances the radiative decay rate of X_D_, without noticeably altering that of X_O_. According to a quantum electrodynamics model based on the Purcell formalism for dissipative cavities, we estimated the intrinsic room-temperature lifetime of X_D_ in a pristine WSe_2_ monolayer to be ~24 ± 2.3 μs. Beyond the lifetime control, we further observed that the X_D_ radiation pattern highly depends on the nanocavity orientation and emitter location, which both govern the Purcell factor distribution within the nanogap and the emitter’s far-field radiation pattern. Tilting the nanocavity allowed the donut-shape radiation pattern to transition into a corner-like shape, demonstrating the capacity to produce a nanoscale light source with high emission directionality. These findings demonstrate an effective approach for leveraging a single plasmonic nanostructure to control the X_D_ emission intensity, dynamics, and radiation directionality. The versatility of this easy-to-implement plasmonic approach, along with the proposed theoretical framework, offers new application prospects for the advancement of optoelectronic devices and quantum information systems based on 2D semiconductors.

## MATERIALS AND METHODS

### Sample fabrication

Gold substrates were prepared by depositing 100-nm-thick gold films at a rate of 0.5 Å/s on a silicon wafer template through electron beam deposition (PVD 75 Thin Film Deposition System, Kurt J. Lesker Company). To obtain a smooth gold film, silica substrates were bonded to the freshly evaporated gold using an ultraviolet (UV)–curable glue and template striped. The TMDC monolayer was mechanically exfoliated from bulk WSe_2_ onto polydimethylsiloxane (PDMS). The monolayer structure was verified with the help of PL spectroscopy. Then, the monolayer WSe_2_ was transferred onto a gold film using the all-dry viscoelastic stamping transfer method (*35**,* *36*). UV light illumination for 20 min was used to eliminate the residual PDMS from the WSe_2_ surface. The Au nanocubes were purchased from X-nanogold Ltd. To achieve stronger film confinement, the cetrimonium bromide protective layer was removed by centrifugation at 2500 rpm for 2 min (*37*). After ultrasonication for 5 min to ensure the uniform separation of single particles, the Au nanocubes were dispersed on the WSe_2_ monolayer using the drop-casting method. The etched WSe_2_-NCoM was obtained from the above structures by directional etching for 25 s using argon plasma bombardment (PVA IoN 7B; 20-W power) to remove WSe_2_ not covered by the nanocubes. The operational pressure was 2 torr, and 2 standard cubic centimeter per minute flow rate of Ar gas was used to generate the plasma for etching.

### Numerical simulation method

Electromagnetic simulations were performed using COMSOL Multiphysics V6.1 software, based on the finite element method. The permittivity of gold was taken from (*38*). A perfectly matched layer was used to enclose the computational domain to minimize unwanted reflections at the domain boundaries. The scattering spectra of the nanocavity were derived by integrating the upward scattering power flow within an angle of 106°, corresponding to the numerical aperture (NA) of 0.8 of the objective lens used in the experiments. The meshing of the simulation models, particularly in the gap region, was carefully refined to ensure computational convergence.

### Coupled oscillator model

LSP mode was modeled as a bright mode that can directly couple with the incident light, while the excitons do not interact with light directly. Assuming no coupling between the two excitons, the plasmon-exciton coupled system can be treated as an external-force–driven oscillator (LSP) coupled with internal oscillators (excitons). In this framework, the light scattering in the two-oscillator system (LSP-exciton) can be described as (*22**,* *39*)

$$\sigma_{\text{sca}}=A\cdot E^{4}{\left|\frac{E^{2}-E_{0}^{2}+i\Gamma_{0}E}{\left(E^{2}-E_{0}^{2}+i\Gamma_{0}E\right)\left(E^{2}-E_{P}^{2}+i\Gamma_{p}E\right)-4\kappa^{2}E^{2}}\right|}^{2}$$ (3)

where *A* is the scattering amplitude; $E_{P}$ and $E_{0}$ are the resonant energies of the gap plasmon mode and bright exciton X_O_; $\Gamma_{p}$ and $\Gamma_{0}$ are the dissipation rate of the gap plasmon mode and X_O_ exciton, respectively; κ is the coupling strength between the gap plasmon mode and X_O_ exciton; and $\sigma_{\text{sca}}$ is the scattering intensity at the energy E.

### Purcell factor calculations with boundary element method

To calculate the spontaneous decay rate of a quantum emitter in an NCoM cavity, we use the MATLAB toolbox MNPBEM to solve the Maxwell’s equations (*28*). A gold nanocube with a size of 85 nm is placed 1.8 nm above a gold mirror. An in-plane (*d_x_*) and out-of-plane (*d_z_*) dipoles are located between the nanocube and the mirror, representing bright and dark excitons, respectively. The total decay rate γ of a quantum emitter in a cavity is expressed as

$$\gamma =\gamma^{\text{free}}+\frac{6\pi \varepsilon_{0}\gamma^{\text{free}}}{{|r|}^{2}k^{3}}\text{Im}\left\{d^{*}\cdot E_{s}\left(r_{0}\right)\right\}$$ (4)

where $\gamma^{\text{free}}$ is the free-space decay rate, ∣r∣ is the distance between the position of the dipole and a reference point in the cavity or environment, k is the wave number, d is the transition dipole moment, and $E_{s}\left(r_{0}\right)$ is the induced secondary electric field at the position of the dipole. In addition, the far-field radiative decay rate $\gamma^{\text{FF}}$ is calculated by integrating the outgoing Poynting vector in the far-field zone over a unit sphere.

### Optical spectroscopy

The dark-field scattering spectra of Au nanocubes were obtained using a home-built multifunctional spectroscopy and imaging system comprising a microscope system (BX53, Olympus) with a 100× dark-field objective, a spectrometer (Shamrock 500i, Andor), and a charge-coupled device (CCD) camera (Newton CCD BEX2-DD, Andor). Individual gold nanocubes were illuminated by a halogen lamp, and the scattered light was collected by the objective for spectral analysis. A standard diffuser is used as a reference to normalize the white light scattering. PL spectra of the pristine WSe_2_ and the WSe_2_-NCoM (Fig. 1) were obtained using a commercial confocal Raman system (WITec Raman alpha300) with a 100× objective (Zeiss LD EC Epiplan-Neofluar Dic; NA = 0.75). A linearly polarized continuous-wave (CW) laser with a wavelength of 532 nm and an intensity of 25 W/cm^2^ was used as the excitation source. TRPL spectra (Fig. 2) were measured using the second harmonic of the Ti: sapphire femtosecond laser (Chameleon Ultra II, Coherent) output at 1064 nm (pulse duration ~ 120 fs) with a repetition rate of 80 MHz (fig. S2). The PL signal from the WSe_2_-NCoM cavity in the focal plane is collected by the same objective. It is then directed into the detection path by a short-pass dichroic mirror, which blocks the pump laser while allowing the PL signal to pass through. A 785 ± 5–nm band-pass filter was used to select the emission of dark excitons. The signal was registered by a single-photon detector [PDM Series, Micro Photon Devices (MPD)]. The output of the MPD was recorded in the PicoHarp 300 system for time-correlated single photon counting measurements. BFP measurements of the PL emission from WSe_2_-NCoM were performed under a 532-nm CW laser excitation through a 100× objective LMPlan with NA = 0.8. To obtain an azimuthally averaged relation between the emission intensity and the polar angle θ in the BFP images, the map was divided into rings corresponding to different θ values. Then, the intensity within each ring was integrated and normalized to the area of the ring

$$I_{\text{average}}\left(\theta \right)=\frac{\int_{0}^{2\pi }\int_{\theta }^{\theta +\Delta \theta }R\;\left(\theta \right)\;I\;\left(\theta ,\varphi \right)\;d\theta d\varphi }{\pi \left[R{\left(\theta +\Delta \theta \right)}^{2}-R{\left(\theta \right)}^{2}\right]}$$ (5)

where R(θ) is the distance between the point and the center of the BFP image and I (θ,φ) is the BFP intensity at the position (θ,φ).

### SEM characterizations

SEM was performed using a Helios 5 CX DualBeam microscope equipped with a field-emission gun. The accelerating voltage was set to 5 kV, and a working distance of ~4 mm was maintained. Samples were mounted with conductive carbon tape to enhance conductivity. Imaging was conducted using a secondary electron detector at a magnification of ×80,000 under high-vacuum conditions.

## Supplementary Materials

### This PDF file includes:

Notes S1 to S3 Figs. S1 to S18 References
