# Supplementary material for: Plasmonic tuning of dark-exciton radiation dynamics and far-field emission directionality in monolayer WSe2
Source: Sci Adv. 2026 Jan 16;12(3):eaea5781. doi: 10.1126/sciadv.aea5781 (PMC12810645; doi:10.1126/sciadv.aea5781)
Supplement: Supplementary file 1 — Notes S1 to S3 Figs. S1 to S18 References [file sciadv.aea5781_sm.pdf]

Science Advances
AAAS

Supplementary Materials for

Plasmonic tuning of dark-exciton radiation dynamics and far-field emission directionality in monolayer WSe_2

Shuaiyu Jin *et al.*

Corresponding author: Dangyuan Lei, dangylei@cityu.edu.hk

Sci. Adv. **12**, eaea5781 (2026)
DOI: 10.1126/sciadv.aea5781

This PDF file includes:

Notes S1 to S3
Figs. S1 to S18
References

Supplementary Text

Note S1: Differential reflectance analysis

Differential reflectance $\Delta R/R$ is linearly related to the sample absorptance A. In a thin film approximation, the connections among reflectance R, transmittance T, and absorptance A at normal incidence for an atomically thin sample on a substrate can be derived from Maxwell's equations (*40*):

$$R = R(\omega, d) = \frac{\left(1 - n_s - \frac{\omega d}{c}\varepsilon_2\right)^2 + \left[\frac{\omega d}{c}(\varepsilon_1 - 1)\right]^2}{\left(1 + n_s + \frac{\omega d}{c}\varepsilon_2\right)^2 + \left[\frac{\omega d}{c}(\varepsilon_1 - 1)\right]^2}, \quad \text{(S1.1 a)}$$

$$T = T(\omega, d) = \frac{4n_s}{\left(1 + n_s + \frac{\omega d}{c}\varepsilon_2\right)^2 + \left[\frac{\omega d}{c}(\varepsilon_1 - 1)\right]^2}, \quad \text{(S1.1 b)}$$

$$A = A(\omega, d) = \frac{4\frac{\omega d}{c}\varepsilon_2}{\left(1 + n_s + \frac{\omega d}{c}\varepsilon_2\right)^2 + \left[\frac{\omega d}{c}(\varepsilon_1 - 1)\right]^2}. \quad \text{(S1.1 c)}$$

Here, n_s is the refractive index of the substrate, ω is the frequency of the incident light, c is the speed of light, d is the effective thickness of the ML WSe_2, and ε_1 and ε_2 are the real and imaginary parts of the sample dielectric function, respectively. For the bare substrate case, $d = 0$, and for the ML WSe_2, d=0.65 nm, we get

$$\Delta R/R = \frac{R - R_s}{R_s} = \left[\left(\frac{n_s + 1}{n_s - 1}\right) + \frac{n_s}{(n_s - 1)^2}\frac{(\gamma_1^2 + \gamma_2^2)}{\gamma_2}\right]A \quad \text{(S1.2)}$$

with $\gamma_1 = \omega d(\varepsilon_1 - 1)/c$ and $\gamma_2 = \omega d\varepsilon_2/c$.

Note S2: Plasmon-exciton coupling regime analysis

In general, the plasmon-exciton hybrid coupling system reaches the strong coupling regime when the sum of the cavity dissipation rate γ_{cav} (experimentally extracted from the linewidth of a cavity scattering spectrum) and the exciton dissipation rate γ_{ex} (experimentally obtained from the exciton PL spectrum) is smaller than twice the coupling strength g between them (*41*):

$$\gamma_{cav} + \gamma_{ex} < 2g. \quad \text{(S2.1)}$$

Conversely, the weak coupling regime is characterized by

$$\gamma_{cav} + \gamma_{ex} > 2g. \quad \text{(S2.2)}$$

In our case, the hybrid WSe_2-NCoM nanocavity involves the coupling between exciton (X_O or X_D) and a gap plasmon mode.

Weak coupling between X_O and a gap plasmon mode

The coupling behavior can be fully described using the coupled-oscillator model, which also allows extraction of the coupling strength g. The largest g determined from the experiment is 0.0483 eV with the detuning: E_P-E_O=13 meV. From the experimental data, we extract $\gamma_{cav} \approx 0.15$ and $\gamma_{ex} \approx 0.03$, clearly satisfying

$$\gamma_{cav} + \gamma_{ex} > 2g, \quad \text{(S2.3)}$$

which confirms that the system is in a weak coupling regime. This conclusion is consistent with the field distribution of the plasmonic mode (Fig. S3), which is mainly oriented along the z-direction, resulting in a weak interaction with the in-plane dipole moment of the X_O excitons.

Furthermore, Fig. S17 shows the energy relation between X_O excitons and the plasmon mode, demonstrating no evidence of Rabi splitting or polaritons, which would be a strong indication of reaching strong coupling regime (*41*, *42*).

Weak coupling between X_D and gap plasmon mode
Since the coupling strength of the X_D excitons cannot be directly extracted experimentally in the same way as for the X_O excitons, we instead rely on indirect evidence. Several experimental observations consistently point to a weak-coupling regime between X_D excitons and the plasmonic modes:

1. Scattering spectrum of WSe_2-NCoM nanocavity: The lack of splitting around the energy of X_D excitons (1.59 eV) implies that no obvious energy exchange occurs between X_D excitons and the gap plasmon mode (*43*). Compared with X_O excitons, X_D-plasmon coupling is even weaker, and the observed emission enhancement can be attributed primarily to the Purcell effect characteristic of a weak coupling rather than to strong coupling.
2. PL emission with different energy detuning: Across different exciton-plasmon detuning conditions, the PL emission spectra of X_D excitons show no peak energy shift. This absence of polariton branches indicates that X_D excitons do not form hybridized polariton states with the cavity.
3. PL lifetime measurements: Time-resolved photoluminescence under various detuning conditions shows radiative decay dynamics that are fully consistent with the Purcell enhancement model. The strong agreement between the experimental lifetime trends and theoretical Purcell predictions provides further confirmation that their interation remains in the weak-coupling regime.

Furthermore, the far-field decay rate exhibits different behavior for emitters with in-plane and out-of-plane dipole orientations (Fig. 3D). This contrast highlights the intrinsically weak transition dipole moment of X_D excitons, which fundamentally limits their coupling strength.

Note S3: Origin of the blue-shift of optimal enhancement point (Fig. 3B) analysis
We first analyze the generalized Purcell formula (*23*):

$$\Gamma = F\Gamma_0 \frac{\omega_0^2}{\omega^2} \frac{\omega_0^2}{\omega_0^2 + 4Q^2(\omega - \omega_0)^2}. \quad \text{(S3.1)}$$

By substituting $\omega_o - \omega$ with x, Eq. S3.1 can be rewritten as

$$Lifetime\left(\frac{1}{\Gamma}\right) = \frac{\omega^2 * [(x+\omega)^2 + 4Q^2x^2]}{F\Gamma_0 * (x+\omega)^4}. \quad \text{(S3.2)}$$

After expanding the numerator, Eq. S3.2 becomes

$$Lifetime\left(\frac{1}{\Gamma}\right) = \frac{\omega^2 * [(4Q+1)(x+\omega)^2 - 8Q^2\omega(x+\omega) + 8Q^2\omega^2 - 4Q^2\omega]}{F\Gamma_0 * (x+\omega)^4}, \quad \text{(S3.3)}$$

which clearly shows that the optimal enhancement point (i.e., the shortest lifetime) is located in this case at $x = 0$ (on resonance).

To further investigate the origin of the experimentally observed blue shift, we extend the model (Eq. 1 in the main text and Eq. S3.1 above) to include the dissipation effects of the nanocavity:

$$Lifetime\left(\frac{1}{\Gamma}\right) = \frac{w^2 * [(x+\omega)^2 + 4Q^2x^2]}{F\Gamma_0 * (x+\omega)^3 * \left\{\left[\mathbf{1} - \mathbf{2Q}\frac{\boldsymbol{Im}(\widetilde{\boldsymbol{V}})}{\boldsymbol{Re}(\widetilde{\boldsymbol{V}})}\right]\boldsymbol{x} + \boldsymbol{\omega}\right\}}. \quad \text{(S3.4)}$$

Eq. S3.4 reveals that the observed blue shift can be attributed to dissipation-induced spectral shifts in the NCoM nanocavity, as discussed in the main text.

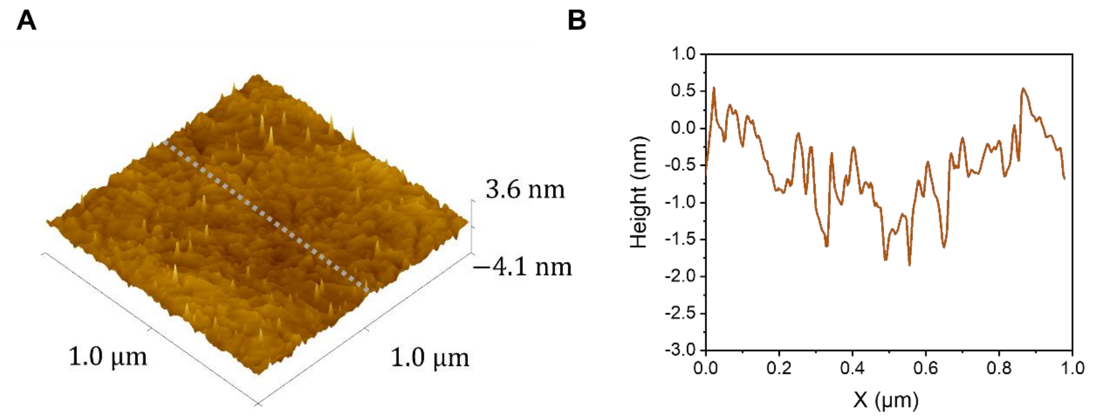

Fig. S1. **Characterization of gold film roughness.** (**A**) 3D Atomic force micrograph of the gold flake deposited at a rate of *0.5* $Å/s$, showing a root mean square (RMS) roughness, $\sigma = \sqrt{\frac{1}{N}\sum_{i=1}^{n}(z_i - \bar{z})^2}$, of approximately 400 pm. (**B**) 2D cross-section view along the dotted line in (A).

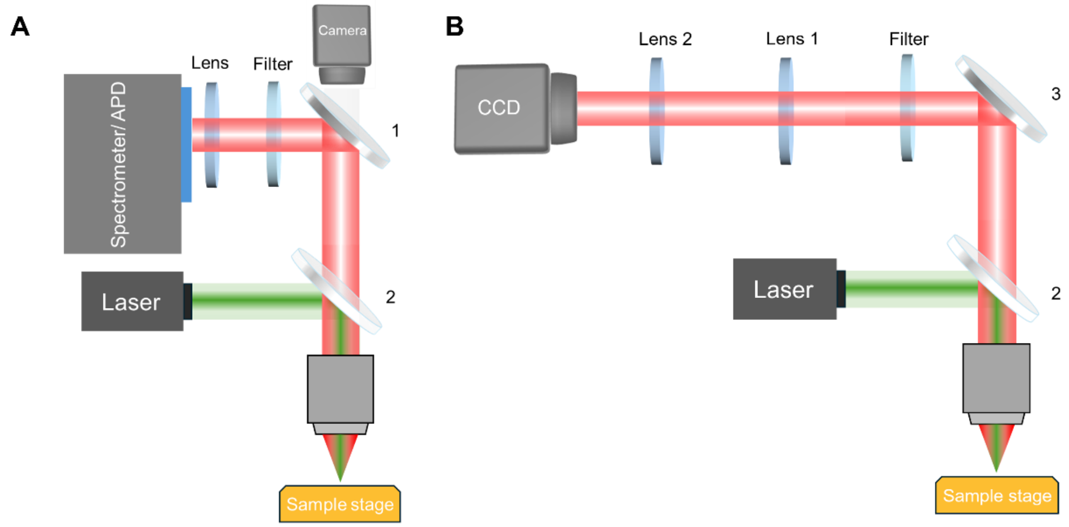

Fig. S2. Experimental setup. (**A**) PL and time-resolved PL spectroscopy and (**B**) back-focal-plane image spectroscopy. 1&2: beam splitter, 3: Silver mirror.

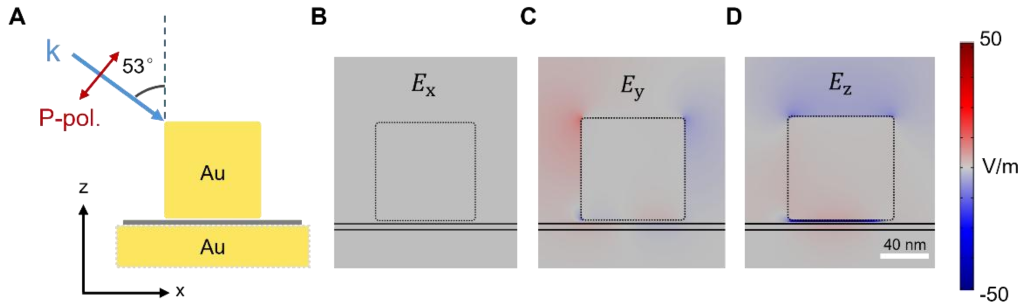

Fig. S3. Simulated electric field distributions of the gap plasmon mode in different directions. (**A**) Schematics of the model for the numerical simulations. Radiation at the 780 nm wavelength is incident at an angle of 53 degrees to the normal direction (*z*-axis). (**B-D**) Electric field distributions of the gap plasmon mode in the NCoM cavity. The field components are plotted in an *xz* plane intersecting the middle of the nanocube. The scale (color bar) is the same for all plots, enabling the comparison of the magnitudes of the electric field components. It is seen that the electric field in the z-direction is strongly enhanced in the gap.

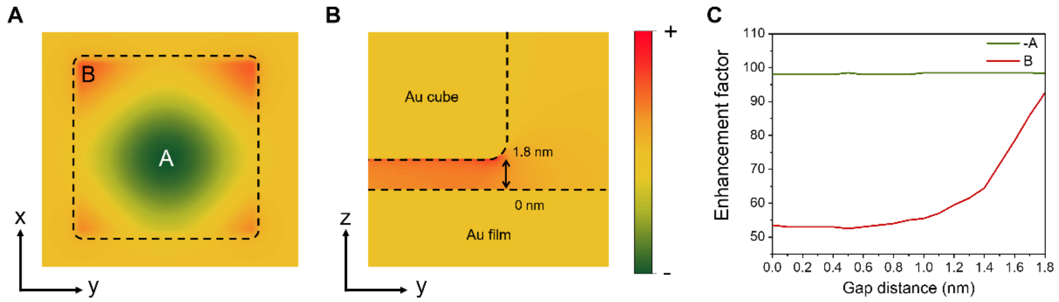

Fig. S4. Simulated electric field distribution of the gap plasmon mode in the gap between the gold nanocube and gold film. (**A**) Calculated map of the out-of-plane electric field component E_z at the gap plasmon mode ($\lambda = 780$ nm) inside the gap at the height of 0.1 nm above the Au mirror. (**B**) Calculated map of the out-of-plane electric field in the vicinity of a corner of the NCoM cavity. (**C**) The out-of-plane electric field at points A (centre) and B (corner) in fig. S4(A) as a function of the height within the gap (0 nm corresponds to the Au film surface).

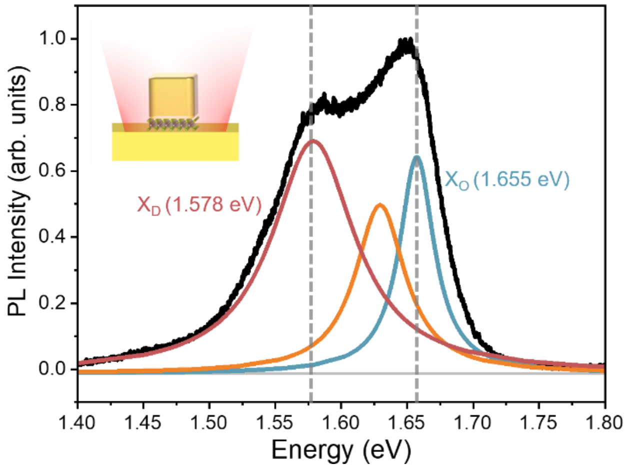

Fig. S5. PL spectrum of the etched WSe_2-NCoM sample. The total spectrum is decomposed into three Lorentzians (solid lines). The emission of X_D exciton (red) is clearly visible. (the inset shows its geometry). The energy difference between the X_D and X_O is ~70 meV, slightly larger than in an unetched sample, which may be attributed to the compressive strain effect after the etching process (*44*).

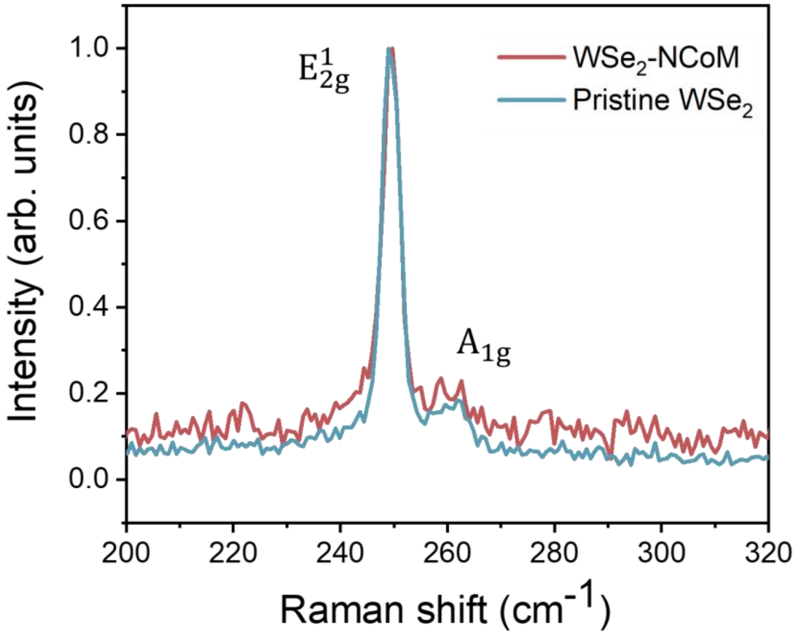

Fig. S6. Raman scattering spectra of pristine WSe_2 and a single WSe_2-NCoM nanocavity. The presence of a 85 nm large nanocube on top of the ML WSe_2 does not alter the position or full width at half maximum (FWHM) of the two phonon modes, indicating that the nanocube does not induce significant strain in the monolayer WSe_2 (*45, 46*).

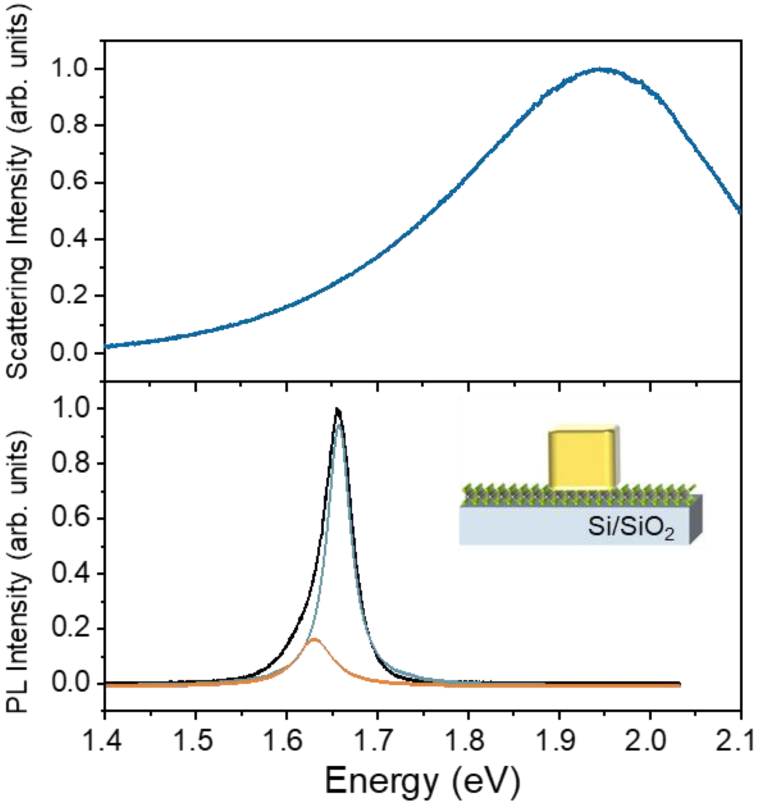

Fig. S7. Scattering and PL spectra of a WSe$_2$-AuNC cavity on a silica substrate. Top: Scattering spectrum of a WSe$_2$-AuNC cavity on a Si/SiO$_2$ substrate. Bottom: PL spectrum of a WSe$_2$-AuNC cavity on a Si/SiO$_2$ substrate, showing a similar peak as pristine ML WSe$_2$. The inset shows the schematics of the corresponding structure.

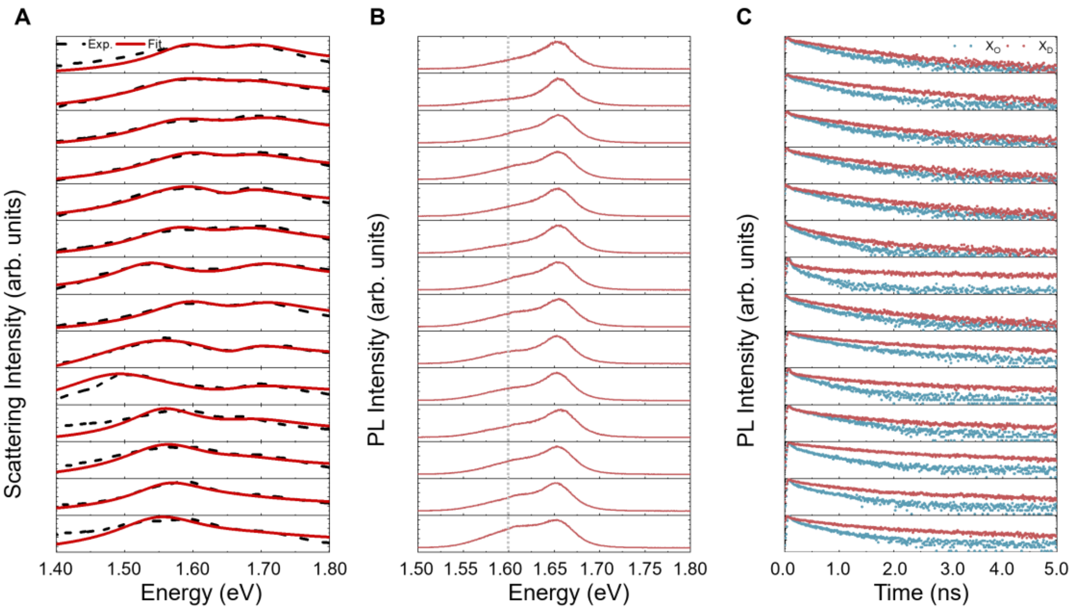

Fig. S8. Spectral measurements of fourteen WSe_2-NCoM nanocavities. (**A**) Scattering spectra, (**B**) PL spectra, and (**C**) TRPL responses of the fourteen WSe_2-NCoM cavities studied in this work. The scattering spectra (dashed lines) were fitted by a coupled oscillator model (solid lines).

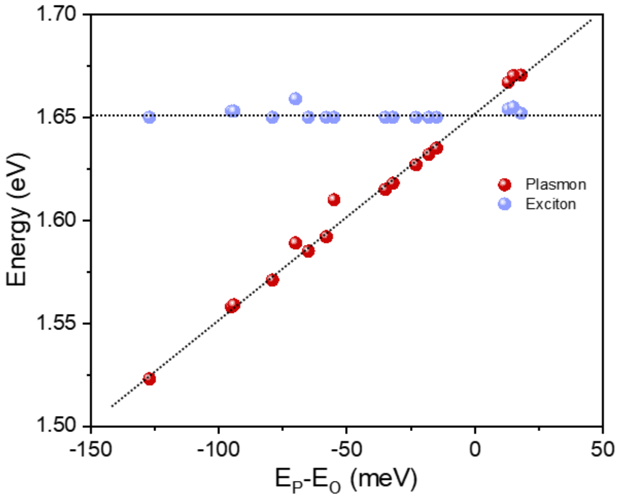

Fig. S9. Energy relation of gap plasmon mode and X_O exciton extracted from the scattering spectrum. This is the evidence of weak coupling between X_O excitons and a gap plasmon mode, as no avoided crossing (Rabi splitting) is observed.

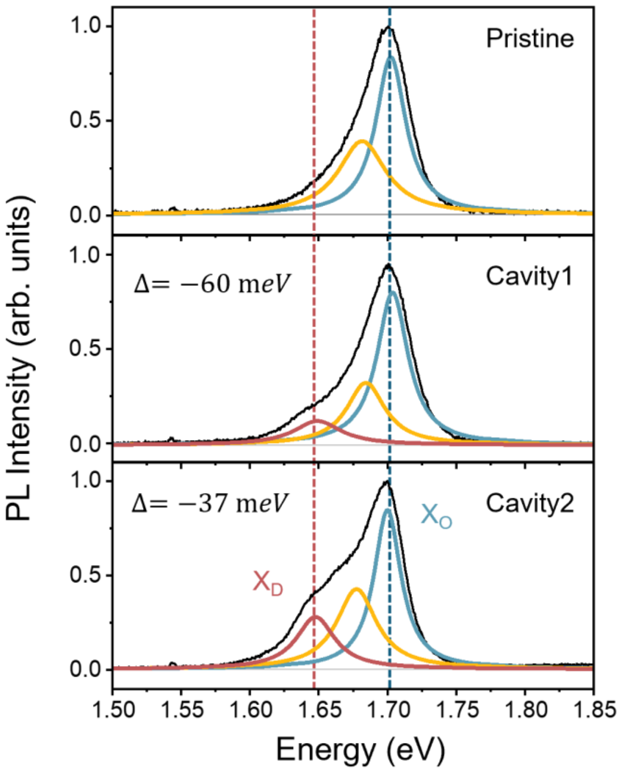

Fig. S10. Low-temperature (150 K) measurements. (Top) The PL spectrum of pristine WSe_2 ML near the WSe_2-NCoM nanocavities, dominated by the emission of X_O and trions. (Middle and bottom) PL spectra of WSe2-NCoM nanocavities with different energy detuning (Δ), as indicated in the panels, between the gap plasmon mode and the X_D exciton, revealing the X_D emission, facilitated by the plasmonic enhancement. The same as at room temperature, the enhancement at low temperatures is still related to the detuning Δ between energy of the gap plasmon mode and X_D. At low temperatures, the lattice contraction of WSe_2 ML reduces the electron-phonon coupling which will further result in an increase in the band gap energy, reflected in the blueshift of exciton peak energy (*47*). Moreover, at low temperatures, the higher-energy phonon population decreases (following the Bose-Einstein distribution), reducing inelastic scattering and causing the PL linewidth narrowing (*48*). The plasmon resonances of NCoM experience no noticeable shifts at low temperatures (*49*, *50*), so that the energy detuning remains almost the same.

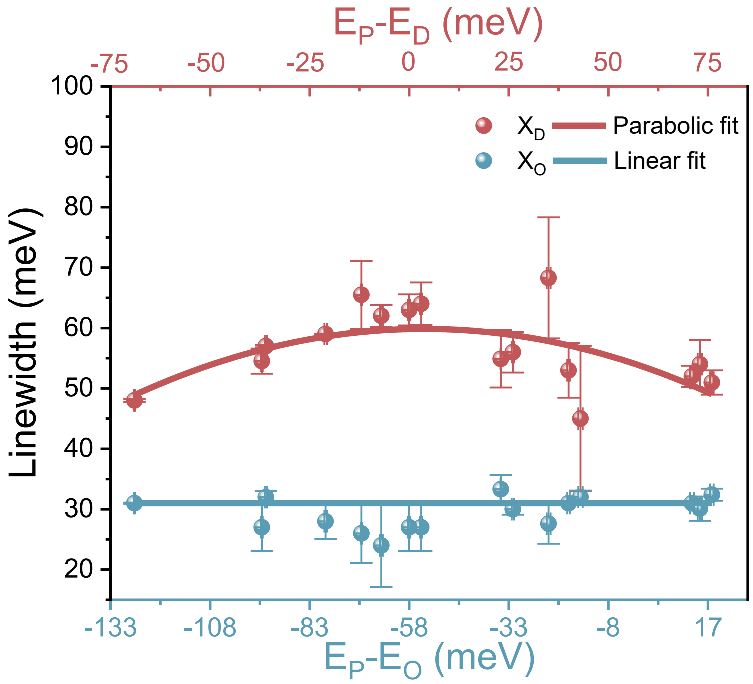

Fig. S11. PL linewidth as a function of energy detuning. The linewidths of X_D (red dots) and X_O (blue dots) excitons in the fourteen studied WSe_2-NCoM cavities as a function of energy detuning between the gap plasmon mode and excitons, showing the parabolic and linear relationships, respectively.

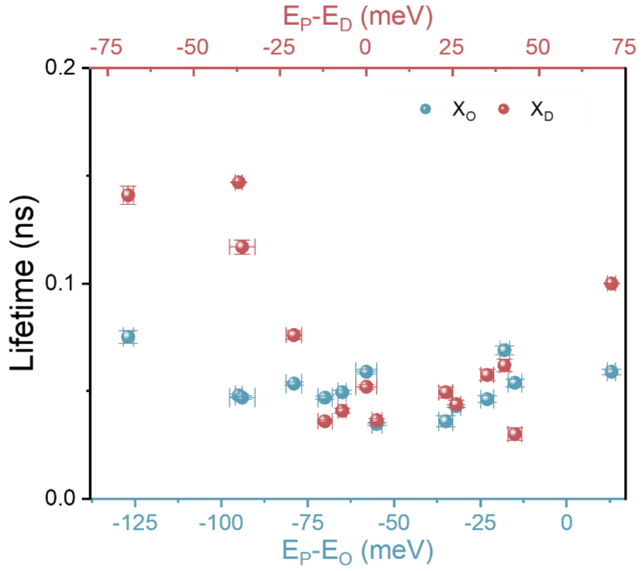

Fig. S12. PL lifetime dependence on detuning. The short lifetime derived from the bi-exponential fitting of X_D and X_O TRPL dependences in the fourteen studied WSe_2-NCoM nanocavities exhibits no clear correlation with the cavity-exciton energy detuning.

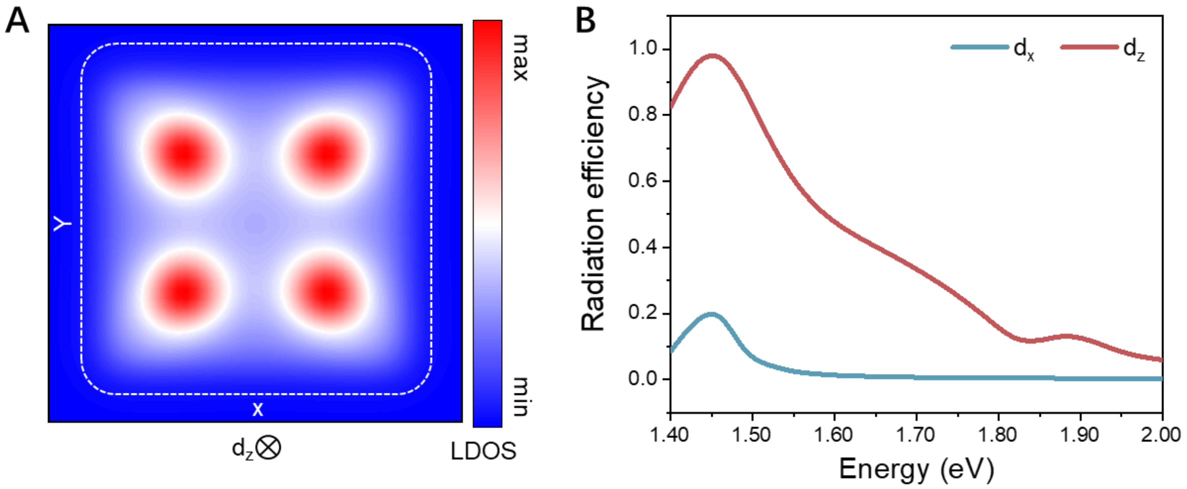

Fig. S13. Spatial distribution of total decay rate enhancement (proportional to the local density of optical states (LDOS)) and radiation efficiency. (**A**) Spatial distribution of the total decay rate enhancement (proportional to the LDOS) at 1.59 eV in a NCoM nanocavity. The maximum enhancement factor of ~60000 is observed at locations close to the four corners of the nanocube. (**B**) The spectrum of radiation efficiency of the in-plane (d_x) and out-of-plane (d_z) dipoles located at the region of the largest Purcell enhancement in the NCoM cavity. Here, the radiation efficiency is defined as Γ_{FF}/Γ which represents the proportion of the enhanced radiative decay rate into free space in the total decay rate. At the emission energy of X_D (1.59 eV), the radiation efficiency is greater than 25%, showing that the intrinsically weak X_D transition can be greatly enhanced in the cavity (*44*).

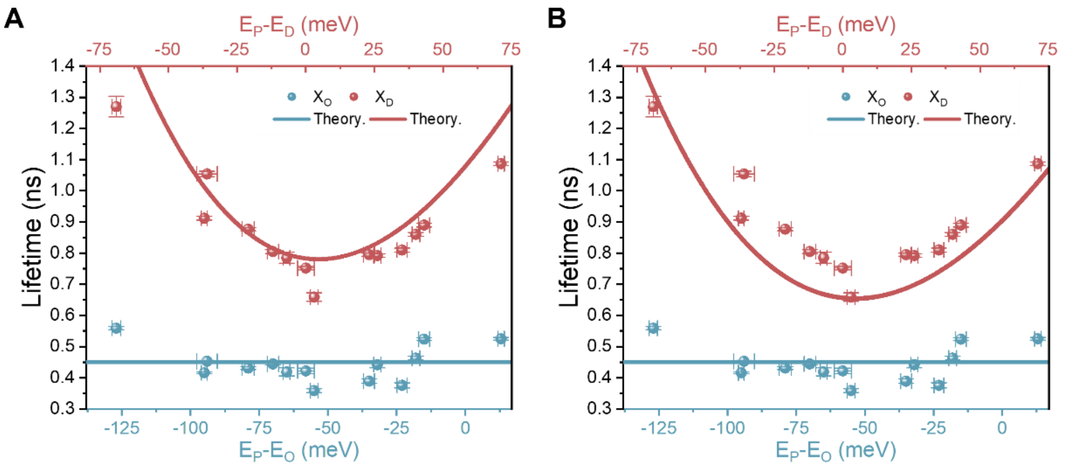

Fig. S14. Uncertainty sources in the estimation of the X_D intrinsic lifetime. (**A**) Results for X_D intrinsic decay rate Γ_0 is 3.8×10^{-5} ns^{-1}, showing the theoretical fit (red solid line) and experimental data (red dots). (**B**) Results for X_D intrinsic decay rate Γ_0 is 4.6×10^{-5} ns^{-1}, with the same representation.

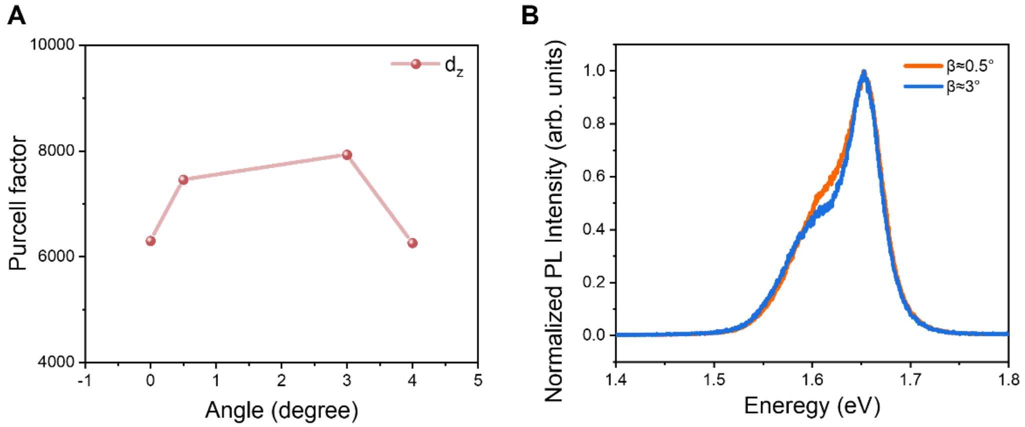

Fig. S15. Effect of cavity tilt angles on X_D PL emission. (**A**) Simulated average Purcell factor in a NCoM nanocavity for four tilted angles. (**B**) PL spectra of the two tilted WSe_2-NCoM nanocavities in Fig. 4. X_D emission is observed irrespective of the tilt angles of the nanocavities. Due to the subtle size variations of individual nanocubes, the plasmon mode energy of the NCoM cavity itself may shift within the range of -75 meV$<E_p - E_D<$75 meV. Thus, it is difficult to identify the origin of the energy shift; nevertheless, the gap plasmon mode energy is still in the required range.

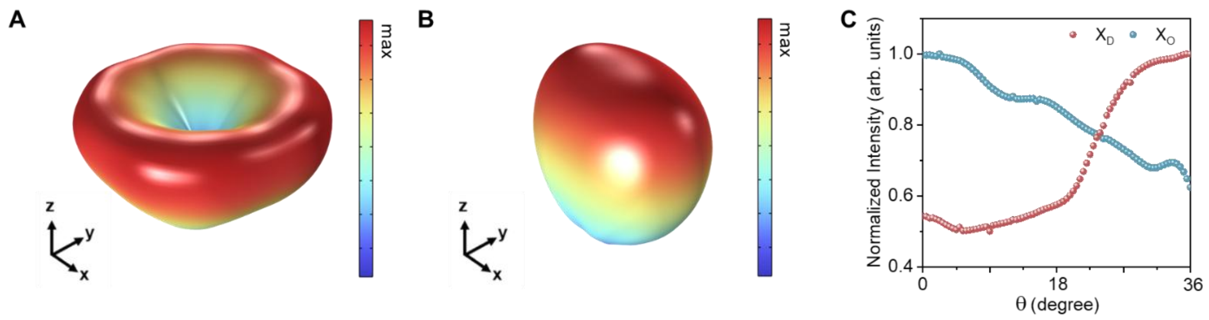

Fig. S16. BFP Comparison between X_D and X_O in WSe_2-NCoM nanocavities. Simulated 3D radiation patterns of the (A) gap mode I and (B) transverse mode III of the NCoM nanocavity. (**C**) Azimuthally averaged angular radiation intensities of X_D and X_O excitons as a function of the polar angle θ in the BFP images, where $\theta = 0^\circ$ corresponds to the center of the BFP image and $\theta = 53^\circ$ corresponds to the objective collection range. The maximum X_D PL intensity occurs at $\sim \pm 36$ degrees. The radiation pattern of the gap plasmon mode has a donut-like shape. At the same time, X_D exciton in the NCoM exhibits an out-of-plane dipole orientation, corresponding to the radiation pattern similar to that of the gap plasmon mode and thus enabling efficient exciton-plasmon coupling. In comparison, the radiation pattern of the transverse mode has an ellipsoidal shape, similar to the emission pattern of an in-plane dipole positioned just above the mirror surface (*51*).

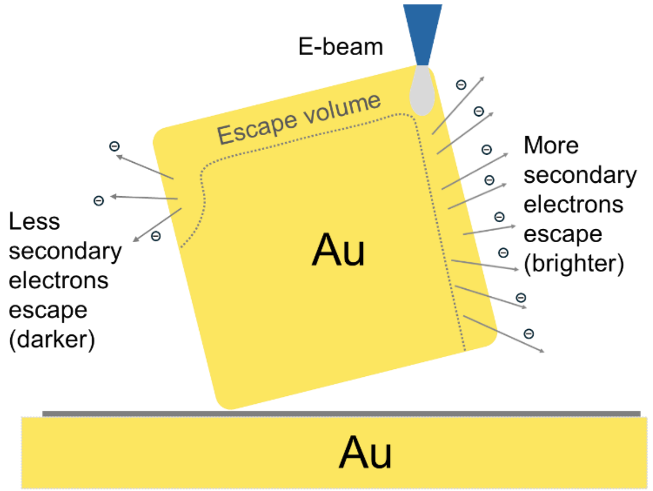

Fig. S17. Illustration of the bright contrast in SEM micrographs of the tilted cavities. The contrast observed between the corners of a nanocube is impacted by the curvature variations. Together with the tilt angle, they significantly alter the escape volume of secondary electrons (SEs) from different regions of the nanocube. The quantity of escaped SEs thus differs across the two sides of a nanocube, resulting in a bright contrast in the SEM image in Fig. 4A.

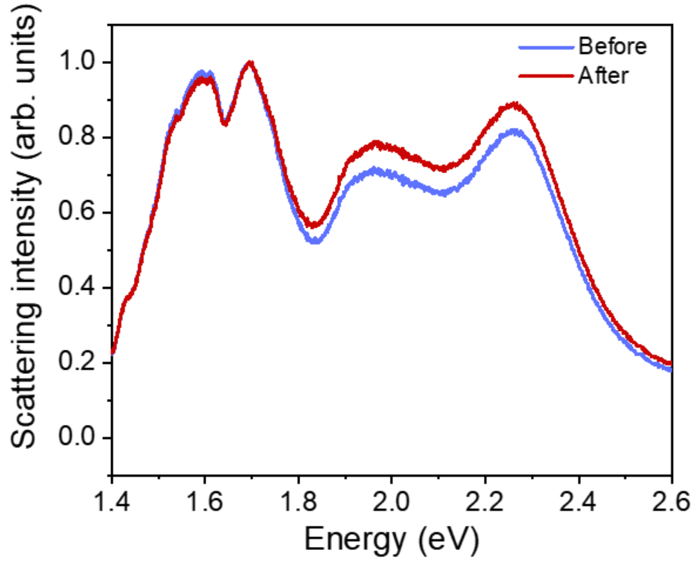

Fig. S18. Comparison of the WSe_2-NCoM nanocavity Scattering spectra before and after measurements. Dark-field scattering spectra of the same WSe_2-NCoM nanocavity, measured before and after TRPL measurements, show negligible alterations in the nanocavity properties during the measurements.

REFERENCES

1. G.-B. Liu, W.-Y. Shan, Y. Yao, W. Yao, D. Xiao, Three-band tight-binding model for monolayers of group-VIB transition metal dichalcogenides. *Phys. Rev. B* **88**, 085433 (2013).

2. K. Kośmider, J. W. González, J. Fernández-Rossier, Large spin splitting in the conduction band of transition metal dichalcogenide monolayers. *Phys. Rev. B* **88**, 245436 (2013).

3. J. P. Echeverry, B. Urbaszek, T. Amand, X. Marie, I. C. Gerber, Splitting between bright and dark excitons in transition metal dichalcogenide monolayers. *Phys. Rev. B* **93**, 121107 (2016).

4. C. T. Yip, T. W. Lo, S.-C. Zhu, G. Y. Jia, H. Sun, C.-H. Lam, D. Lei, Tight-binding modeling of excitonic response in van der Waals stacked 2D semiconductors. *Nanoscale Horiz.* **4**, 969–974 (2019).

5. X.-X. Zhang, Y. You, S. Y. F. Zhao, T. F. Heinz, Experimental evidence for dark excitons in monolayer WSe_2. *Phys. Rev. Lett.* **115**, 257403 (2015).

6. M. R. Molas, C. Faugeras, A. O. Slobodeniuk, K. Nogajewski, M. Bartos, D. M. Basko, M. Potemski, Brightening of dark excitons in monolayers of semiconducting transition metal dichalcogenides. *2D Mater.* **4**, 021003 (2017).

7. T. Smoleński, T. Kazimierczuk, M. Goryca, P. Wojnar, P. Kossacki, Mechanism and dynamics of biexciton formation from a long-lived dark exciton in a CdTe quantum dot. *Phys. Rev. B* **91**, 155430 (2015).

8. K.-D. Park, T. Jiang, G. Clark, X. Xu, M. B. Raschke, Radiative control of dark excitons at room temperature by nano-optical antenna-tip Purcell effect. *Nat. Nanotechnol.* **13**, 59–64 (2018).

9. C. Robert, T. Amand, F. Cadiz, D. Lagarde, E. Courtade, M. Manca, T. Taniguchi, K. Watanabe, B. Urbaszek, X. Marie, Fine structure and lifetime of dark excitons in transition metal dichalcogenide monolayers. *Phys. Rev. B* **96**, 155423 (2017).

10. X.-X. Zhang, T. Cao, Z. Lu, Y.-C. Lin, F. Zhang, Y. Wang, Z. Li, J. C. Hone, J. A. Robinson, D. Smirnov, S. G. Louie, T. F. Heinz, Magnetic brightening and control of dark excitons in monolayer WSe2. *Nat. Nanotechnol.* **12**, 883–888 (2017).

11. G. Wang, C. Robert, M. M. Glazov, F. Cadiz, E. Courtade, T. Amand, D. Lagarde, T. Taniguchi, K. Watanabe, B. Urbaszek, X. Marie, In-plane propagation of light in transition metal dichalcogenide monolayers: Optical selection rules. *Phys. Rev. Lett.* **119**, 047401 (2017).

12. D. J. Roth, P. Ginzburg, L. M. Hirvonen, J. A. Levitt, M. E. Nasir, K. Suhling, D. Richards, V. A. Podolskiy, A. V. Zayats, Singlet–triplet transition rate enhancement inside hyperbolic metamaterials. *Laser Photonics Rev.* **13**, 1900101 (2019).

13. Y. Zhou, G. Scuri, D. S. Wild, A. A. High, A. Dibos, L. A. Jauregui, C. Shu, K. De Greve, K. Pistunova, A. Y. Joe, T. Taniguchi, K. Watanabe, P. Kim, M. D. Lukin, H. Park, Probing dark excitons in atomically thin semiconductors via near-field coupling to surface plasmon polaritons. *Nat. Nanotechol.* **12**, 856–860 (2017).

14. T. W. Lo, X. Chen, Z. Zhang, Q. Zhang, C. W. Leung, A. V. Zayats, D. Lei, Plasmonic nanocavity induced coupling and boost of dark excitons in monolayer WSe_2 at room temperature. *Nano Lett.* **22**, 1915–1921 (2022).

15. Q. H. Wang, K. Kalantar-Zadeh, A. Kis, J. N. Coleman, M. S. Strano, Electronics and optoelectronics of two-dimensional transition metal dichalcogenides. *Nat. Nanotechnol.* **7**, 699–712 (2012).

16. Z. Ye, T. Cao, K. O'Brien, H. Zhu, X. Yin, Y. Wang, S. G. Louie, X. Zhang, Probing excitonic dark states in single-layer tungsten disulphide. *Nature* **513**, 214–218 (2014).

17. Z. Wang, L. Liu, D. Zhang, A. V. Krasavin, J. Zheng, C. Pan, E. He, Z. Wang, S. Zhong, Z. Li, M. Ren, X. Guo, A. V. Zayats, L. Tong, P. Wang, Effect of mirror quality on optical response of nanoparticle-on-mirror plasmonic nanocavities. *Adv. Opt. Mater.* **11**, 2201914 (2023).

18. X. Li, L. Zhou, Z. Hao, Q. Wang, Plasmon–exciton coupling in complex systems. *Adv. Opt. Mater.* **6**, 1800275 (2018).

19. N. S. Mueller, R. Arul, G. Kang, A. P. Saunders, A. C. Johnson, A. Sánchez-Iglesias, S. Hu, L. A. Jakob, J. Bar-David, B. De Nijs, L. M. Liz-Marzán, F. Liu, J. J. Baumberg, Photoluminescence upconversion in monolayer WSe_2 activated by plasmonic cavities through resonant excitation of dark excitons. *Nat. Commun.* **14**, 5726 (2023).

20. O. A. Ajayi, J. V. Ardelean, G. D. Shepard, J. Wang, A. Antony, T. Taniguchi, K. Watanabe, T. F. Heinz, S. Strauf, X.-Y. Zhu, J. C. Hone, Approaching the intrinsic photoluminescence linewidth in transition metal dichalcogenide monolayers. *2D Mater.* **4**, 031011 (2017).

21. H. Wei, X. Yan, Y. Niu, Q. Li, Z. Jia, H. Xu, Plasmon–exciton interactions: Spontaneous emission and strong coupling. *Adv. Funct. Mater.* **31**, 2100889 (2021).

22. T. W. Lo, Q. Zhang, M. Qiu, X. Guo, Y. Meng, Y. Zhu, J. J. Xiao, W. Jin, C. W. Leung, D. Lei, Thermal redistribution of exciton population in monolayer transition metal dichalcogenides probed with plasmon–exciton coupling spectroscopy. *ACS Photonics* **6**, 411–421 (2019).

23. C. Sauvan, J. P. Hugonin, I. S. Maksymov, P. Lalanne, Theory of the spontaneous optical emission of nanosize photonic and plasmon resonators. *Phys. Rev. Lett.* **110**, 237401 (2013).

24. T. Wu, M. Gurioli, P. Lalanne, Nanoscale light confinement: The Q's and V's. *ACS Photonics* **8**, 1522–1538 (2021).

25. G.-H. Jung, S. Yoo, Q.-H. Park, Measuring the optical permittivity of two-dimensional materials without a priori knowledge of electronic transitions. *Nanophotonics* **8**, 263–270 (2019).

26. M. Palummo, M. Bernardi, J. C. Grossman, Exciton radiative lifetimes in two-dimensional transition metal dichalcogenides. *Nano Lett.* **15**, 2794–2800 (2015).

27. A. Lombardi, A. Demetriadou, L. Weller, P. Andrae, F. Benz, R. Chikkaraddy, J. Aizpurua, J. J. Baumberg, Anomalous spectral shift of near- and far-field plasmonic resonances in nanogaps. *ACS Photonics* **3**, 471–477 (2016).

28. J. Waxenegger, A. Trügler, U. Hohenester, Plasmonics simulations with the MNPBEM toolbox: Consideration of substrates and layer structures. *Comput. Phys. Commun.* **193**, 138–150 (2015).

29. Z. Sun, J. Gu, A. Ghazaryan, Z. Shotan, C. R. Considine, M. Dollar, B. Chakraborty, X. Liu, P. Ghaemi, S. Kéna-Cohen, V. M. Menon, Optical control of room-temperature valley polaritons. *Nat. Photonics* **11**, 491–496 (2017).

30. S. I. Bogdanov, M. Y. Shalaginov, A. S. Lagutchev, C.-C. Chiang, D. Shah, A. S. Baburin, I. A. Ryzhikov, I. A. Rodionov, A. V. Kildishev, A. Boltasseva, V. M. Shalaev, Ultrabright room-temperature sub-nanosecond emission from single nitrogen-vacancy centers coupled to nanopatch antennas. *Nano Lett.* **18**, 4837–4844 (2018).

31. H. Chen, Z. Jiang, H. Hu, B. Kang, B. Zhang, X. Mi, L. Guo, C. Zhang, J. Li, J. Lu, L. Yan, Z. Fu, Z. Zhang, H. Zheng, H. Xu, Sub-50-ns ultrafast upconversion luminescence of a rare-earth-doped nanoparticle. *Nat. Photonics* **16**, 651–657 (2022).

32. X. Qi, T. W. Lo, D. Liu, L. Feng, Y. Chen, Y. Wu, H. Ren, G.-C. Guo, D. Lei, X. Ren, Effects of gap thickness and emitter location on the photoluminescence enhancement of monolayer MoS_2 in a plasmonic nanoparticle-film coupled system. *Nanophotonics* **9**, 2097–2105 (2020).

33. M. J. Horton, O. S. Ojambati, R. Chikkaraddy, W. M. Deacon, N. Kongsuwan, A. Demetriadou, O. Hess, J. J. Baumberg, Nanoscopy through a plasmonic nanolens. *Proc. Natl. Acad. Sci. U.S.A.* **117**, 2275–2281 (2020).

34. S. Hu, E. Elliott, A. Sánchez-Iglesias, J. Huang, C. Guo, Y. Hou, M. Kamp, E. S. A. Goerlitzer, K. Bedingfield, B. De Nijs, J. Peng, A. Demetriadou, L. M. Liz-Marzán, J. J. Baumberg, Full control of plasmonic nanocavities using gold decahedra-on-mirror constructs with monodisperse facets. *Adv. Sci.* **10**, e2207178 (2023).

35. J. Sun, H. Hu, D. Zheng, D. Zhang, Q. Deng, S. Zhang, H. Xu, Light-emitting plexciton: Exploiting plasmon–exciton interaction in the intermediate coupling regime. *ACS Nano* **12**, 10393–10402 (2018).

36. H. Gao, Y. Hu, Y. Xuan, J. Li, Y. Yang, R. V. Martinez, C. Li, J. Luo, M. Qi, G. J. Cheng, Large-scale nanoshaping of ultrasmooth 3D crystalline metallic structures. *Science* **346**, 1352–1356 (2014).

37. G.-C. Li, Q. Zhang, S. A. Maier, D. Lei, Plasmonic particle-on-film nanocavities: A versatile platform for plasmon-enhanced spectroscopy and photochemistry. *Nanophotonics* **7**, 1865–1889 (2018).

38. P. B. Johnson, R. W. Christy, Optical constants of the noble metals. *Phys. Rev. B* **6**, 4370–4379 (1972).

39. D. Zheng, S. Zhang, Q. Deng, M. Kang, P. Nordlander, H. Xu, Manipulating coherent plasmon–exciton interaction in a single silver nanorod on monolayer WSe_2. *Nano Lett.* **17**, 3809–3814 (2017).

40. E. J. Sie, A. Steinhoff, C. Gies, C. H. Lui, Q. Ma, M. Rösner, G. Schönhoff, F. Jahnke, T. O. Wehling, Y.-H. Lee, J. Kong, P. Jarillo-Herrero, N. Gedik, Observation of exciton redshift–blueshift crossover in monolayer WS_2. *Nano Lett.* **17**, 4210–4216 (2017).

41. R. Chikkaraddy, B. De Nijs, F. Benz, S. J. Barrow, O. A. Scherman, E. Rosta, A. Demetriadou, P. Fox, O. Hess, J. J. Baumberg, Single-molecule strong coupling at room temperature in plasmonic nanocavities. *Nature* **535**, 127–130 (2016).

42. I. A. M. Al-Ani, K. As'ham, O. Klochan, H. T. Hattori, L. Huang, A. E. Miroshnichenko, Recent advances on strong light-matter coupling in atomically thin TMDC semiconductor materials. *J. Opt.* **24**, 053001 (2022).

43. J. Cuadra, D. G. Baranov, M. Wersäll, R. Verre, T. J. Antosiewicz, T. Shegai, Observation of tunable charged exciton polaritons in hybrid monolayer WS_2 − Plasmonic nanoantenna system. *Nano Lett.* **18**, 1777–1785 (2018).

44. G.-H. Peng, P.-Y. Lo, W.-H. Li, Y.-C. Huang, Y.-H. Chen, C.-H. Lee, C.-K. Yang, S.-J. Cheng, Distinctive signatures of the spin- and momentum-forbidden Dark exciton states in the

photoluminescence of strained WSe_2 monolayers under thermalization. *Nano Lett.* **19**, 2299–2312 (2019).

45. A. M. Dadgar, D. Scullion, K. Kang, D. Esposito, E. H. Yang, I. P. Herman, M. A. Pimenta, E.-J. G. Santos, A. N. Pasupathy, Strain engineering and raman spectroscopy of monolayer transition metal dichalcogenides. *Chem. Mater.* **30**, 5148–5155 (2018).

46. Z. Peng, X. Chen, Y. Fan, D. J. Srolovitz, D. Lei, Strain engineering of 2D semiconductors and graphene: From strain fields to band-structure tuning and photonic applications. *Light Sci. Appl.* **9**, 190 (2020).

47. S. Rai, A. Srivastava, Low-temperature photoluminescence and Raman study of monolayer WSe2 for photocarrier dynamics and thermal conductivity. *J. Appl. Phys.* **136**, 154301 (2024).

48. M. Manca, M. M. Glazov, C. Robert, F. Cadiz, T. Taniguchi, K. Watanabe, E. Courtade, T. Amand, P. Renucci, X. Marie, G. Wang, B. Urbaszek, Enabling valley selective exciton scattering in monolayer WSe_2 through upconversion. *Nat. Commun.* **8**, 14927 (2017).

49. M. Liu, M. Pelton, P. Guyot-Sionnest, Reduced damping of surface plasmons at low temperatures. *Phys. Rev. B* **79**, 035418 (2009).

50. X. Xia, H. Huang, S. Li, R. Ai, Z. Tian, F. Yan, L. Shao, J. Wang, Switching on dark excitons in WSe_2 monolayer at room temperature with plasmonic au nanodisk-on-mirror cavities. *Laser Photonics Rev.* **19**, e02012 (2025).

51. Q. Zhang, G.-C. Li, T. W. Lo, D. Y. Lei, Polarization-resolved optical response of plasmonic particle-on-film nanocavities. *J. Opt.* **20**, 024010 (2018).
